# CpG-Recoding in Zika Virus Genome Causes Host-Age-Dependent Attenuation of Infection With Protection Against Lethal Heterologous Challenge in Mice

**DOI:** 10.3389/fimmu.2019.03077

**Published:** 2020-01-24

**Authors:** Ivan Trus, Daniel Udenze, Nathalie Berube, Colette Wheler, Marie-Jocelyne Martel, Volker Gerdts, Uladzimir Karniychuk

**Affiliations:** ^1^Vaccine and Infectious Disease Organization-International Vaccine Centre (VIDO-InterVac), University of Saskatchewan, Saskatoon, SK, Canada; ^2^School of Public Health, University of Saskatchewan, Saskatoon, SK, Canada; ^3^Department of Obstetrics and Gynecology, College of Medicine, University of Saskatchewan, Saskatoon, SK, Canada; ^4^Department of Veterinary Microbiology, Western College of Veterinary Medicine, University of Saskatchewan, Saskatoon, SK, Canada

**Keywords:** CpG dinucleotide, Zika virus, neonate, adult, age, vaccine, ZAP

## Abstract

Experimental increase of CpG dinucleotides in an RNA virus genome impairs infection providing a promising approach for vaccine development. While CpG recoding is an emerging and promising vaccine approach, little is known about infection phenotypes caused by recoded viruses *in vivo*. For example, infection phenotypes, immunogenicity, and protective efficacy induced by CpG-recoded viruses in different age groups were not studied yet. This is important, because attenuation of infection phenotypes caused by recoded viruses may depend on the population-based expression of cellular components targeting viral CpG dinucleotides. In the present study, we generated several Zika virus (ZIKV) variants with the increasing CpG content and compared infection in neonatal and adult mice. Increasing the CpG content caused host-age-dependent attenuation of infection with considerable attenuation in neonates and high attenuation in adults. Expression of the zinc-finger antiviral protein (ZAP)—the host protein targeting viral CpG dinucleotides—was also age-dependent. Similar to the wild-type virus, ZIKV variants with the increased CpG content evoked robust cellular and humoral immune responses and protection against lethal challenge. Collectively, the host age should be accounted for in future studies on mechanisms targeting viral CpG dinucleotides, development of safe dinucleotide recoding strategies, and applications of CpG-recoded vaccines.

## Introduction

Dinucleotide frequencies deviate from what is randomly expected (1); the most striking example is the suppression of cytosine-phosphate-guanine (CpG) dinucleotide frequencies in vertebrates and most vertebrate RNA viruses (2, 3). Experimental increase of CpG dinucleotides in a relatively small region of an RNA viral genome, while retaining the amino acid composition of encoded proteins, impairs infection *in vitro* and *in vivo* (4–9) providing a promising approach for live modified vaccines. Cytosine-phosphate-guanine dinucleotide recoding is an emerging vaccine approach, so little is known yet about infection phenotypes caused by recoded viruses *in vivo* and in different population and age groups.

The traditional rationale of CpG dinucleotide suppression by the methylation-deamination-mutation hypothesis cannot explain why RNA viruses have an underrepresentation of CpG dinucleotides similar to vertebrates (10). But there are important milestones in understanding the mechanisms of CpG-mediated impairment of viral infection (4–7, 11, 12). First, the CpG-impaired infection phenotype arises through more efficient targeting of the recoded viruses by host immunity rather than replicative defectiveness of the mutants (4, 13). Second, CpG mutants can enter cells, but restrictive host responses act shortly after entry with incoming virions failing to form replication complexes (12). Third, restriction of infection is not mediated through translation impairment, disruption of an RNA structure, or stress, interferon, and apoptosis pathways activated through conventional pattern recognition receptors (4, 10–12). Finally, zinc-finger antiviral protein (ZAP) targets recoded human immunodeficiency virus 1 (HIV-1) and echovirus 7 by directly binding to CpG-enriched genomic regions (9, 14); subsequently, synergy or complementation of ZAP function by oligoadenylate synthetase 3, RNase L (15) and cytoplasmic protein KHNYN (16) is capable of inhibiting replication of viruses containing the elevated number of CpG dinucleotides.

In addition to the intriguing questions about virus-host interactions, the rational increase of CpG dinucleotide numbers may become a cutting-edge approach and alternative to traditional live attenuated vaccines (LAVs) (4, 7, 17). LAVs capitalize on single-dose immunization, robust immune responses, and long-lasting protection. The most successful examples of partial (e.g., poliomyelitis, rubella virus) and full (smallpox) eradication of devastating human infections are attributed to LAVs. However, the traditional development of LAVs is associated with time-consuming attenuation in cell cultures, uncontrollable generation of a small number of random mutations responsible for attenuation, and safety issues due to the potential for reversion of attenuated strains to the virulent phenotype. CpG-recoded vaccine candidates are also capable of replicating, but in contrast to traditional LAVs, where typically few substitutions induce virus attenuation—e.g., attenuated oral poliovirus vaccine Sabin strains have only a single mutation critical for attenuation (18)—this technology is based on the cumulative effect of many nucleotide mutations resulting in hundreds of additional CpG dinucleotides. Each additional CpG dinucleotide may have a contributing impact, potentially providing a tunable approach to impairing viral infection to the desired degree, minimizing reversion to the virulent state, and optimizing vaccine safety and efficacy (4). Importantly, in contrast to the lengthy classical attenuation process, CpG recoding utilizes *de novo* gene synthesis and reverse genetics and may become a fast, adaptable vaccine technology for rapid responses to emerging pathogens.

Attenuated infection caused by recoded vaccine candidates may depend on the expression of cellular components targeting CpG dinucleotides (15); thus, focused investigations on population-based differences in CpG-recoded vaccine attenuation to reassure safety and efficacy are crucial (7, 15). In this context, comparative studies in different age-groups are required for basic knowledge of emerging CpG-recoding vaccine technology; this basic knowledge may determine future rational applications of CpG-recoded vaccines in animals and humans.

In the present study, we worked with Zika virus (ZIKV) as a model because it causes infection in hosts of different age—neonates and adults (19, 20). And animal models for neonatal and adult ZIKV infection are well-established (21–24). We generated several ZIKV variants with the increased CpG and normalized uracil-phosphate-adenine (UpA) genomic content. First, infection phenotypes of CpG-recoded variants were compared in cell lines and primary human cells. We also compared the stability of *de novo* introduced CpG dinucleotides during *in vitro* and *in vivo* infections. Second, we compared infection phenotypes and immunogenicity in neonatal and adult animal models. Third, we quantified expression of ZAP—the host factor targeting viral genomic CpG dinucleotides—in tissues of fetuses, neonates, and adults in health and during infection. Finally, we assessed whether immunization of mice with ZIKV-recoded variants protects against heterologous lethal challenge.

## Materials and Methods

### Cell Lines

RD cells (ATCC CCL-136) were maintained in Dulbecco's modified Eagle's medium (DMEM; Sigma D5796) supplemented with 10% fetal bovine serum (FBS; Sigma 12103c) and 1x Penicillin-Streptomycin (Gibco 15140-122). VERO E6 cells (ATCC CRL-1586) were maintained in DMEM supplemented with 3% FBS, 1x Penicillin-Streptomycin and 2.67 mM Sodium Bicarbonate (Gibco 25080-094). HTR-8/SVneo (ATCC CRL-3271) were maintained in Roswell Park Memorial Institute 1640 Medium (RPMI; Gibco 11875119) supplemented with 5% FBS and 1x Penicillin-Streptomycin. C6/36 cells (ATCC CRL-1660) were maintained in Minimum Essential Medium (MEM; Sigma M4655) supplemented with 10% FBS and 1x Penicillin-Streptomycin. RD, VERO, HTR-8/SVneo cells were cultured at +37°C (C6/36 cells were cultured at +28°C) in a 5% CO_2_ humidified incubator.

### Generation of Human Monocyte-Derived Dendritic Cells (moDCs) and Flow Cytometry Analysis

Human peripheral blood (10–20 ml) was collected from nine healthy, hepatitis B, C, and HIV-negative adults (eight females and one male) by venipuncture into an EDTA-containing vacuum tube (BD Vacutainer). Blood was diluted 1:2 in Dulbecco's phosphate-buffered saline (DPBS; Gibco 14190-144) and peripheral blood mononuclear cells were isolated by centrifugation over Lymphocyte Separation Medium (Corning 25-072-CV) followed by three washes in DPBS. To isolate CD14+ monocytes, we used positive immunomagnetic bead selection according to the manufacturer's instructions (Miltenyi Biotec CD14 MicroBeads and LS Columns). Monocytes were seeded in 24-well flat bottomed plates at a density of 2 × 10^6^ cells/ml in RPMI 1640 Medium supplemented with 10% FBS, 1x Penicillin-Streptomycin and 50 μg/ml Gentamycin (Gibco 15750-060). To generate moDCs, cells were supplemented with 100 ng/ml GM-CSF and 100 ng/ml IL-4 (Miltenyi Biotec 130-093-866 and 130-093-922) and cultured at +37°C in a 5% CO_2_ humidified incubator for 7 days. After the first 12 h of incubation, non-adherent cells and medium were gently removed and fresh medium was added. On day 3 and 5, half of the culture media was replaced with fresh media. At day 7, non-adherent cells were collected and used for subsequent infection experiments as described below.

Flow cytometry was performed on a CyAn ADP flow cytometer with Summit 4.4 software (Beckman Coulter). Data were analyzed by Kaluza 2.1 software (Beckman Coulter). The cell surface expression of moDC specific phenotype markers was assessed on day 0 and 7. Cells were first incubated for 20 min on ice with 20 μl of Fc receptor binding inhibitor (Life Technologies LS14916173) and then stained with the fluorochrome-conjugated antibodies (Abs) against CD11 (BD 562561) and CD14 (BD 561116) on ice for 45 min. The phenotype of moDCs after the infection was assessed using a cocktail containing Abs against HLA-DR (BD 555812), CD40 (BD 563396), CD80 (BD 563315), and CD86 (BD 561129). Dead cells were excluded from the analysis using LIVE/DEAD Fixable Near-IR Dead Cell Stain Kit (Life Technologies LSL34975) according to the manufacturer's protocol.

### *In silico* Design of CpG-Recoded ZIKV Variants

**Figure 1:**
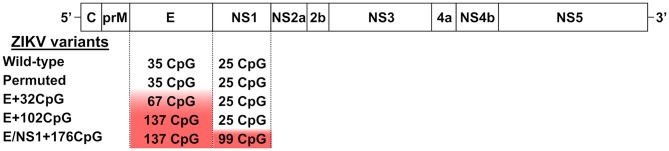
The genome of ZIKV and the recoding strategy. ZIKV genomic regions encoding E and NS1 proteins were recoded to increase the number of CpG dinucleotides. The actual number of CpG dinucleotides in the ZIKV genomic regions is denoted.

Zika virus has a single plus-strand RNA genome with a single open reading frame (ORF) encoding a polyprotein that is subsequently cleaved by cellular and viral proteases into three structural proteins (C, prM, and E) and seven nonstructural proteins (NS1, NS2A, NS2B, NS3, NS4A, NS4B, and NS5) (25, 26). For recoding, we selected genomic regions encoding E and NS1 because these proteins are important for ZIKV pathogenesis, modulation of the infection cycle, viral RNA replication, and host immune evasion (27–29). We used a SSE 1.3 software package (30); the MUTATE SEQUENCES program in the SSE package allows CpG recoding without affecting protein sequences, mononucleotide composition, and with minor effects on codon parameters (4, 5, 11). The sequence of the contemporary Asian ZIKV H/PF/2013 strain [GenBank: KJ776791.2] was used as a wild-type (WT) prototype (31). We recoded the wild-type ZIKV genome sequence to generate E+32CpG, E+102CpG, and E/NS1+176CpG variants with increased CpG numbers in E and NS1 (Figure 1). We sought to avoid areas of the genome containing RNA elements that are required for replication or translation of the virus genome, such as *cis*-replicating elements, gene start or gene-end signals; we also avoided regions with prominent secondary structures (5).

The number of additional CpG dinucleotides in the panel of ZIKV variants ranges from 32 in the E+32CpG variant to 176 in the E/NS1+176CpG variant (Figure 1). Using the MUTATE SEQUENCES program, we renormalized UpA dinucleotides in all CpG variants to the original level because increasing UpA dinucleotides also reduces viral fitness (4, 5, 11). To ensure sequence disruption did not damage or destroy the undetected replication element(s), we designed a permuted control. In the control, the sequence region was permuted using the CDLR method in the SSE software package (4, 5, 11); this randomizes the order of codons within the sequence while maintaining coding parameters and dinucleotide frequencies.

### Recovery of CpG-Recoded ZIKV Variants

To recover ZIKV variants we used infectious subgenomic amplicons (ISA) (31–33) as previously described (32). For the WT ZIKV variant, three pUC57 plasmids [EVAg 001N-01891] containing overlapping regions (PF-I: 1-3428 nt, PF-II: 3354-7621 nt, PF-III: 7553-10807; all nucleotide (nt) positions based on complete viral strain sequence) covering the full ZIKV H/PF/2013 genome were used (31). Regions encoding envelope (E) and non-structural 1 (NS1) proteins within the original fragment PF-I were recoded to increase CpG dinucleotide frequencies (Figure 1). Recoded PF-I fragments were synthesized, and sequences were verified and cloned into the pCC1 vector (GenScript, http://www.genscript.com). Overlapping DNA fragments were amplified with high fidelity PCR (Invitrogen Platinum PCR SuperMix, High Fidelity), transfected into C6/36 *Aedes albopictus* mosquito cells at +37°C for 12 h, and incubated for 7 days at +28°C (32). Media from virus-negative C6/36 cells was used as a control for transfection.

After passaging twice in C6/36 cells, cell culture media containing ZIKV was centrifuged (12,000 g, 20 min, +4°C), and frozen (−80°C). Viral titers were quantified in triplicates in VERO cells with endpoint dilution assay described below. The absence of mycoplasma contamination in all virus stocks and cell cultures was confirmed using PCR Detection Kit (Sigma MP0035).

### *In vitro* and *in vivo* Stability of *de novo* Introduced CpG Dinucleotides in Recoded ZIKV Variants

To study the stability of recoded sequences *in vitro*, we passaged ZIKV variants (Figure 1) ten times in VERO cells. Cells were seeded in 24-well plates 24 h before virus inoculation at multiplicity of infection (MOI) of 0.01. After 1 h at +37°C, the inoculum was discarded and cells were washed three times with +37°C DPBS. Fresh culture medium was added and cells were incubated for 7 days at +37°C, 5% CO_2_. Afterward, the supernatant was collected and used to inoculate cells for the next passage. After 10 passages, the supernatant was collected for RNA extraction (see below) and reverse transcription (Invitrogen SuperScript IV Reverse Transcriptase). The CpG enriched segments were amplified (Invitrogen Platinum PCR SuperMix, High Fidelity) and sequenced (Applied Biosystems ABI-3730xL platform) using both amplification and inner nested primers listed in Supplementary Table 1. Sequences from the 10th passage were compared to sequences from the reference virus stocks obtained on C6/36 cells at passage 2 after transfection.

For the *in vivo* stability assay, RNA was extracted from brains of C57BL/6 mice inoculated at one day of age with different ZIKV variants (Figure 1) and sampled at 21 days after inoculation (see neonatal mouse study). The CpG enriched segments were amplified and sequenced using both amplification and inner nested primers listed in Supplementary Table 2.

### *In vitro* Replication Phenotypes of CpG-Recoded ZIKV Variants

We evaluated the viral replication kinetics in RD, HTR-8/SVneo, VERO, C6/36, and human moDCs. Cells in suspension were inoculated at MOI of 0.01 (for moDCs MOI = 1) in 100 μl of appropriate cell culture medium. Eppendorfs with inoculated cells were incubated at +37°C for 1 h and shaken gently every 10 min. Afterward, cells were washed three times with media and seeded in 96-well plates. Wells were first prefilled with 150 μl of cell culture medium and 50 μl of cell suspension was added on top to get a resulting concentration of 4 × 10^4^ cells per well (5 × 10^4^ cells per well for moDCs). Plates corresponding to one experimental time point were infected with ZIKV variants at the same initial time. Mock-infected cells were included as controls in each plate.

Infected plates were incubated (5% CO_2_, +37°C) until the sampling time point when supernatants were collected, clarified (2,000 g, 5 min) and frozen (−80°C) for subsequent infectious virus quantification (34–37). After the supernatant collection, the plate with infected cells was dried and frozen (−20°C). Plates were stained with virus-specific 4G2 Abs (36), and infected cells were counted in the well with bright-field microscopy at 200x magnification and expressed per cm^2^. Cell culture supernatants and fixed plates were collected daily at 0–5 days post inoculation (or at other time points indicated in figure legends), with three technical replicates and three biological replicates per time point for each ZIKV variant.

### *In vitro* Competition Assay With ZIKV Variant-Specific Restriction Digestion

An *in vitro* competition assay (4, 5, 11, 38, 39) compares the relative replicative fitness of any two ZIKV variants (Figure 1) after co-infection and serial passage in susceptible cells. Briefly, 4 × 10^5^ VERO cells were seeded in 24-well plates. After 24 h, medium was removed and inoculum (0.3 ml) containing equal MOI (0.01 each) of two different ZIKV variants was added to cells. After 2 h, the inoculum was removed, cells were washed four times with DPBS and fresh medium was added. At 7 days post-incubation, 50 μl of supernatant was diluted 6-fold and used for inoculation (2 h) of fresh cells for the next passage. Five passages were done in triplicate for each ZIKV variant pair. After five passages, supernatants were collected and centrifuged (2,000 g, 5 min, +4°C), extracted RNA was reverse transcribed (Invitrogen PureLink RNA Mini Kit and Invitrogen SuperScript IV Reverse Transcriptase), and a specific 1.6 kb region was amplified (Invitrogen Platinum PCR SuperMix, High Fidelity) by a primer pair that anneals equally to both virus variants in each pair (ZIKV-F919 and ZIKV-R2539 listed in Supplementary Table 2). For variant pairs containing the E/NS1+176CpG mutant, a longer fragment (2.6 kb) was amplified using the alternative primer set (ZIKV-F919 and ZIKV-R3498 listed in Supplementary Table 1). The amplified fragment was then subjected to restriction cleavage with endonuclease enzymes (NEB) that only digest amplicon of one of the two mixed ZIKV variants (*PstI* for the permuted ZIKV variant, *BsaBI* for E+32CpG, *XhoI* for E+102CpG, and *SacII* for E/NS1+176CpG). Cleaved and intact PCR products had a different size after separation with agarose gel electrophoresis (Supplementary Figure 1). Digestion profiles of competing viruses in each virus combination were compared after the fifth passage; digestion profiles of not-mixed ZIKV variants and mixed ZIKV variants before passage in VERO cells were included as controls.

### Animal Experiments

We used the second passage of ZIKV variants on C6/36 cells for all inoculations.

To compare infection phenotypes and immune responses induced by different ZIKV variants in neonates (24), and safety of potential vaccine candidates in neonates, timed-pregnant C57BL/6 mice were assigned to groups. After birth, 1 day-old pups were inoculated intraperitoneally (IP) (BD 320468, “Ultra-Fine II 30G 5/16” Syringe) with 10^2^ or 10^4^ TCID_50_ of different ZIKV variants (Figure 1) in 50 μl of DPBS. Control animals were mock-inoculated with virus-free media. Neonates were observed daily for neurological clinical signs. At 21 days after inoculation, mice were anesthetized with isoflurane for cardiac puncture and blood sampling. Blood in EDTA tubes was clarified by centrifugation (2,000 g, +4°C, 20 min) and stored at −80°C for serological assays. Brains from all animals were sampled and tested for ZIKV loads and in immunohistochemistry as described below.

To compare infection phenotypes and immune responses induced by different ZIKV variants in adults, and safety of potential vaccine candidates in adults during pregnancy, we used the pregnancy model (22, 40). Eight- to 10 weeks-old BALB/c female mice were mated to 12 week-old males in our animal facilities. At embryonic day 5 (E5), plugged dams were injected IP with 2 mg of anti-IFNAR1 Abs (Leinco MAR1-5A3) (22). At E6, mice were inoculated subcutaneously (SC) with 10^4^ TCID_50_ of different ZIKV variants or control virus-free media. Animals were sacrificed at E18, and placentas, fetal heads, uterus, maternal spleen, and brains were harvested to quantify virus loads. Maternal blood plasma was also collected for serological assays. All fetuses from each dam were photographed for quantification and examination.

To compare ZAP expression in different age-groups cryosections from fetal (eight fetal heads from three control mice), neonatal (eleven neonatal brains from control animals), and adult brains (samples from eleven 8- to 10-weeks-old mice) were stained and analyzed as described below.

To compare cellular and humoral immune responses induced by different ZIKV variants, 12-week-old BALB/c male mice were assigned into four groups and injected IP with 2 mg of anti-IFNAR1 Abs. On the next day, mice were immunized SC with 10^4^ TCID_50_ of different ZIKV variants or control virus-free media. At 0 and 28 days after inoculation, blood plasma was collected for serology and spleen (at 28 days) for ELISpot assay as described below.

To assess the protective efficacy of different ZIKV variants, 12-weeks-old BALB/c male mice were vaccinated SC with 10^5^ TCID_50_ (in 100 μl DPBS) of different ZIKV variants or control virus-free media. All vaccinations were performed 1 day after the IP administration of 2 mg of anti-IFNAR1 Abs. At 0 and 28 days after vaccination, blood plasma samples were collected for serological assays. At 38 days, mice were challenged intracerebrally (23) with 10^5^ TCID_50_ (in 30 μl DPBS) of heterologous mouse-adapted African MR-766 ZIKV strain [GenBank: KU963573.2, BEI Resource Repository: NR-50065]; historically, the challenge virus was passaged 150 times in suckling mice. Clinical observations and bodyweight measurements were obtained daily. Blood samples were collected and mice were sacrificed when exhibiting >20% weight loss or at the end of the study−14 days after challenge. The right brain hemisphere and spleen were preserved at −80°C for virus load quantification and immunohistochemistry. The left brain hemisphere was preserved in 10% buffered formalin for hematoxylin and eosin (H&E) staining and histopathological examinations.

### RNA Extraction and Reverse Transcriptase Quantitative Polymerase Chain Reaction Assay (RT-qPCR)

The lateral surface of the right cerebral hemisphere and cerebellum in neonatal and adult mice were shaved with a sterile scalpel blade to collect 20–40 mg of tissue. The whole fetal heads in mice were used for RNA extraction. Tissue samples were weighed on analytical balances, 1 ml of TRI Reagent Solution (Invitrogen AM9738) was added, and tissues were homogenized using RNase-free stainless steel beads and TissueLyser II (QIAGEN) operating for 5 min at 25 Hz. Then RNA extraction was performed with PhaseMaker tubes (Invitrogen A33248) and PureLink RNA Mini Kit (Invitrogen 12183025) according to the manufacturer's instructions. For mouse placental and spleen tissues, PureLink RNA Mini Kit was used to extract RNA according to the manufacturer's instructions.

PCR reactions were conducted on the StepOne Plus platform (Life Technologies) and analyzed using StepOne 2.3 software. ZIKV specific SYBRgreen-based one-step RT-qPCR was used for ZIKV RNA quantification (41). The reaction mixture (20 μl) consisted of 10 μl 2x SensiFAST SYBR Hi-ROX One-Step Mix (Bioline BIO-73005), 0.4 μl RiboSafe RNase Inhibitor, 0.2 μl reverse transcriptase, 0.8 μl (400 nM) of each primer (ZIKV-F10287: 5′-AGGATCATAGGTGATGAAGAAAAGT-3′; ZIKV-R10402: 5′-CCTGACAACACTAAGATTGGTGC-3′), 3.8 μl nuclease-free water and 4 μl RNA template. A reverse transcription step of 10 min at 45°C and an enzyme activation step of 2 min at 95°C were followed by 40 amplification cycles (5 s at 95°C and 34 s at 60°C). RNA (10238-1044 = 207 nt amplicon) from a ZIKV stock was used to generate a standard curve and quantify viral RNA loads. The results indicated that the standard curve had a wide dynamic range (10^2^−10^9^ copies/reaction) with the high linear correlation (*R*^2^ = 0.9997) between the cycle threshold (Cq) value and template concentration. The slope of the standard curve (−3.4351) corresponded to the 95.5% reaction efficiency level. PCR values were corrected for fluid volumes or tissue weights and upon logarithmical transformation expressed as ZIKV RNA genome copies per ml or gram. In all PCR tests, we used VERO cell culture media containing ZIKV as a positive PCR control. As a negative control, we used samples from mock-inoculated control animals. Strict precautions were taken to prevent PCR contamination. Aerosol-resistant filter pipette tips and disposable gloves were used. Kit reagent controls were included in every RNA isolation and PCR run.

### Virus Titration

For quantification of infectious ZIKV loads in cell culture media the endpoint dilution assay was used as described before (34–37, 42). Cell culture media were serially diluted 5-fold in four replicates starting from 1:5, and 50 μl of each dilution was added to confluent VERO cells cultured in 96-well plates. Dilutions were made in DMEM media. After 2 h of incubation, 150 μl of fresh media was added to each well. The cells were incubated for 7 days. After washing and drying, the plates were kept at −20°C at least for 2 h or until use. Cell fixation and staining were done, as previously described (34–37, 42); anti-pan flavivirus E protein monoclonal Abs 4G2 (ATCC HB-112) were used to detect ZIKV-infected cells. Fifty percent endpoint titers were calculated by the Spearman-Kärber formula and expressed in a decimal logarithm of TCID_50_. Media from mock-inoculated cells were used as negative controls.

### ELISpot Assay

Isolation and stimulation of splenocytes were performed as previously described (43, 44). Freshly isolated mouse splenocytes (10^6^ cells/well) were seeded in RPMI supplemented with 10% FBS, 1x MEM Non-Essential Amino Acids Solution (Gibco 11140-050) 1x Penicillin-Streptomycin, 50 μg/ml Gentamycin, 10 mM HEPES (MP Biomedicals 1688449), 1 mM Sodium Pyruvate (Gibco 11360-070), and 50 μM 2-mercaptoethanol and stimulated (20 h, +37°C, 5% CO_2_) with a pool of 146 15-meric overlapping peptides derived from E protein of ZIKV (JPT PM-ZIKV-E, 2 μg/ml per peptide). Interferon γ antigen secreting cells (IFNγ-SC) were quantified in duplicates using a murine IFNγ ELISpot kit (Diaclone 862.031.001S) and the *i*Spot Reader System (AID ELR07IFL), according to the manufacturer's instructions. The background level of cellular responses measured in wells with non-stimulated splenocytes was subtracted in all processed samples.

### Serology

For quantification of ZIKV-specific IgG Ab in mouse blood plasma, an adapted immunoperoxidase monolayer assay (IPMA) was used as described before (34–37, 42). Briefly, VERO cells in 96-well cell culture plates were inoculated with 50 μl media containing 5 TCID_50_ of ZIKV and incubated for 2 h (+37°C, 5% CO_2_). Then 100 μl of the culture medium (DMEM supplemented with 5% FCS, 1x Penicillin-Streptomycin, 2.67 mM Sodium Bicarbonate) was added and after incubation (72 h, +37°C, 5% CO_2_) plates were dried and stored at −20°C until use. Plates were thawed and cells were fixed in 10% buffered formalin (30 min, RT). Cells were washed twice with 1x DPBS (pH 7.2) and incubated with 100% methanol in the presence of 0.3% H_2_O_2_ (10 min, RT). Then plates were washed with DPBS and 2-fold serial dilutions of blood plasma were added, followed by 1 h incubation (+37°C). Plates were washed three times with DPBS containing 0.05% Tween-80 and 50 μl/well of goat anti-mouse IgG conjugated with horseradish peroxidase (1:2000, Abcam ab97023) was added. After incubation (1 h, +37°C) and washing, the color reaction was initiated by adding substrate solution (1 mM 3-amino-9-ethylcarbazole, 5% N,N-dimethylformamide, 50 mM Sodium Acetate (pH 5.0), 10 mM H_2_O_2_). The reaction was stopped by replacing the substrate with an acetate buffer and ZIKV-specific staining was determined by examination with a microscope. The Ab titers were defined as the log reciprocal of the highest serum dilution. Blood plasma from mock-inoculated control animals was used as a negative control.

### Virus-Neutralizing (VN) Assay

We quantified ZIKV-neutralizing Abs in mouse blood plasma with an adapted virus-neutralizing assay as described before (34, 35). Briefly, 50 μl of ZIKV (100 TCID_50_/ml) were mixed with equal volumes of 2-fold serially diluted plasma (in two replicates) and incubated at +37°C for 1 h before inoculation VERO cells in 96-well plates. After 2 h, 100 μl/well of fresh DMEM supplemented with 1% FBS, 1x Penicillin-Streptomycin and 2.67 mM Sodium Bicarbonate was added. After 5 days cells were fixed and stained with ZIKV-specific 4G2 Abs as described for virus titration. The neutralizing Ab titers were expressed as the log reciprocal of the highest plasma dilution that inhibited ZIKV infection in 50% of the inoculated wells. Blood plasma from mock-inoculated control animals was used as a negative control.

### Histopathology and Immunohistochemistry

Mouse brain tissues were dissected and fixed in formalin for subsequent H&E staining to screen lesions (45, 46). For immunohistochemistry, staining was performed as previously described (36, 47) with some modifications. For CD68, mouse brain cryosections of 12 μm were fixed in 10% buffered formalin for 15 min at +4°C for 15 min. After treatment with 0.3% H_2_O_2_ and 1% Triton X-100 (10 min, RT), tissue sections were incubated with rat IgG2a monoclonal Abs (Biolegend, 137001; dilution 1:100) against mouse CD68 for 1 h at +37°C. Afterward, the sections were incubated with rabbit anti-rat IgG HRP Abs (Abcam, Ab6734; 1:300) following Lab Vision Ready-To-Use AEC Substrate System (Thermo Fisher Scientific) according to the manufacturer's instructions. Subsequently, tissues were counterstained with hematoxylin and sections were analyzed with a light microscope. For ZAP, brain (head for fetuses) cryosections of 12 μm were fixed and permeabilized as above, incubated with rabbit polyclonal anti-mouse ZAP Abs (Proteintech, 16820-1-AP, 1:50) and visualized with Mouse and Rabbit Specific HRP/DAB IHC Detection Kit—Micro-polymer (ab236466) according to manufacturer's instructions and counterstained with hematoxylin. The entire tissue sections were scanned using Aperio ScanScope CS2 (Leica Biosystems) at a scanning magnification of x20, and images were analyzed using Aperio Image Scope v12.1.0.5029 software.

### Statistical Analysis

We used GraphPad PRISM 8 software. Results were considered to be significantly different when *p* < 0.05. All data were expressed as mean ± standard deviation (M ± SD). In all *in vitro* and *in vivo* experiments, groups infected with modified ZIKV variants were compared vs. WT ZIKV.

ZIKV loads in cell culture (RD, HTR-8/Vneo, C6/36, and VERO) supernatants were subjected to analysis of variance for repeated measures (rANOVA). The number of cells (RD, HTR-8/Vneo, and VERO) infected with different ZIKV variants were compared using rANOVA after shifted log-transformation [ln(x+1)]. To compare ZIKV infection and expression of activation markers in moDCs we fitted a mixed-effects model.

Neurological clinical scores in neonatal mice were compared using rANOVA. Survival curves were compared with the Mantel-Cox log-rank test. The number of dead mouse fetuses was compared with Yates-corrected χ^2^-test. In the protective efficacy mouse study, bodyweight dynamics were compared using a mixed-effects model. ZIKV loads in tissues, IFNγ-SC counts, and Ab titers in mouse studies were compared with Kruskal-Wallis *H*-test and Dunn's multiplicity-adjusted post-test.

ZAP expression in different age groups was compared with the Yates-corrected χ^2^-test.

In all comparisons involving rANOVA and mixed-effects model, the Geisser-Greenhouse correction was applied before carrying out the Dunnett's post-test.

## Results

### Transfection With CpG-Recoded Sub-genomic Amplicons Results in Infectious ZIKV Variants

**Figure 2:**
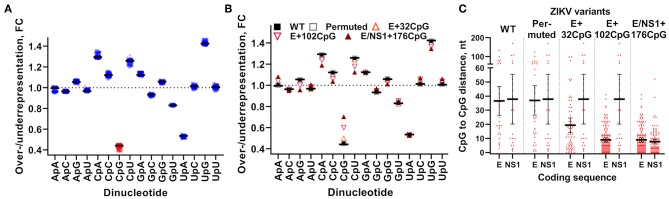
Dinucleotide composition in ZIKV variants. CpG dinucleotide suppression in **(A)** complete wild-type ZIKV genomes (*n* = 773) retrieved from GenBank on 1.10.2019 and **(B)** CpG-recoded ZIKV variants. Over- or underrepresentation (fold change, FC) was calculated for each dinucleotide: observed prevalence [p_observed_(CpG)] was divided by estimated frequency [p_estimated_(CpG)] based on mononucleotides occurrence across the whole genome—e.g., p_estimated_(CpG) = p(C) × p(G). Solid lines represent mean. Dotted lines represent expected level of dinucleotide representation. **(C)** The density of CpG dinucleotides within ZIKV E and NS1 coding regions. Y-axis represents a number of nucleotides in between two neighboring CpGs. Solid lines and whiskers represent mean and 95% confidence intervals.

We used previously described dinucleotide recoding principles (5) and infectious subgenomic amplicons (33, 48) to generate infectious ZIKV variants. *In silico* analysis demonstrated that WT ZIKV genomes, as expected, had suppression of CpG dinucleotides (Figure 2A). We increased CpG dinucleotide frequencies in genomic regions encoding E and NS1 proteins (Figure 2B; Supplementary Figure 2); protein-coding sequences remained unaltered. We also renormalized frequencies of UpA dinucleotides in recoded ZIKV variants to the initial level (Table 1). In addition to the increased CpG dinucleotide numbers, recoded ZIKV variants had denser CpG allocation (Figure 2C). Recoded variants showed a modest reduction in codon pair bias scores in the E and NS1 genomic regions or minimal changes in the complete ORF (Table 2).

**Table 1:** CpG and UpA dinucleotide composition in ZIKV variants.

**Genomic region**	**ZIKV variants**	**Substitutions^a^**	**Number of CpGs**	**Relative overrepresentation of CpG dinucleotides^b^**	**O/E CpG ratio^c^**	**Number of UpAs**	**Relative overrepresentation of UpA dinucleotides^b^**	**O/E UpA ratio^c^**
E	Wild-type	–	35	1.00	0.38	43	1.00	0.46
	Permuted	195	35	1.00	0.38	43	1.00	0.46
	E+32CpG	135	67	1.91	0.73	42	0.98	0.45
	E+102CpG	280	137	3.91	1.48	43	1.00	0.46
	E/NS1+176CpG	280	137	3.91	1.48	43	1.00	0.46
NS1	Wild-type	–	25	1.00	0.40	34	1.00	0.53
	Permuted	0	25	1.00	0.40	34	1.00	0.53
	E+32CpG	0	25	1.00	0.40	34	1.00	0.53
	E+102CpG	0	25	1.00	0.40	34	1.00	0.53
	E/NS1+176CpG	176	99	3.96	1.51	28	0.82	0.45
ORF^d^	Wild-type	-	316	1.00	0.45	337	1.00	0.53
	Permuted	195	316	1.00	0.45	337	1.00	0.53
	E+32CpG	135	348	1.10	0.50	336	1.00	0.53
	E+102CpG	280	418	1.32	0.60	337	1.00	0.53
	E/NS1+176CpG	456	492	1.56	0.70	331	0.98	0.53

a *Number of nucleotide changes vs. the WT ZIKV variant*.

b *Relative overrepresentation vs. the WT ZIKV variant*.

c *Ratio of observed dinucleotide frequency to estimated (O/E ratio)*.

d *ORF, open reading frame*.

**Table 2:** Genomic analysis of CpG-recoded ZIKV variants.

**Genome region**	**Sequence**	**G+C content, %**	**GC3, %**	**ENc^a^**	**CAI^b^**	**CPB**^c^ **(human)**
E	Wild-type	49.8	53.0	52.7	0.756	0.032
	Permuted	49.8	53.0	53.9	0.759	0.020
	E+32CpG	49.9	52.6	54.6	0.728	−0.022
	E+102CpG	49.9	50.6	48.4	0.639	−0.132
	E/NS1+176CpG	49.9	50.6	48.4	0.639	−0.132
NS1	Wild-type	50.1	53.4	51.5	0.784	0.001
	Permuted	50.1	53.4	51.5	0.784	0.001
	E+32CpG	50.1	53.4	51.5	0.784	0.001
	E+102CpG	50.1	53.4	51.5	0.784	0.001
	E/NS1+176CpG	50.8	51.1	51.5	0.705	−0.193
ORF^d^	Wild-type	51.1	55.1	53.4	0.755	0.021
	Permuted	51.1	55.1	53.6	0.755	0.019
	E+32CpG	51.1	55.0	53.9	0.751	0.013
	E+102CpG	51.1	54.7	55.4	0.736	−0.003
	E/NS1+176CpG	51.2	54.5	56.3	0.728	−0.023

a *ENc—Effective Number of Codons (49)*.

b *CAI—Codon Adaptation Index (50)*.

c *Codon pair bias was calculated using codon pair scores from Kunec and Osterrider (7)*.

d *ORF, open reading frame*.

Transfection of 1 μg of an equimolar mixture of three overlapping subgenomic amplicons representing ZIKV variants (Supplementary Figure 3) in mosquito C6/36 cells resulted in the recovery of infectious ZIKV variants. The mosquito genome does not show underrepresentation of CpG dinucleotides (7). Also, in C6/36 cells, all CpG-recoded ZIKV variants had similar infection kinetics compared to the WT variant (Supplementary Figure 4). This makes mosquito cells suitable for recovery and growth of viruses with increased CpG content. In support, after two passages in C6/36 cells all variants showed infectious virus titers expressed with TCID_50_: WT: 10^7.7^ TCID_50_/ml; Permuted: 10^6.8^ TCID_50_/ml; E+32CpG: 10^7.0^ TCID_50_/ml; E+102CpG: 10^7.0^ TCID_50_/ml; and NS1/E+176CpG: 10^6.6^ TCID_50_/ml.

Collectively, using rational CpG dinucleotide recoding, simple reverse genetics method, and transfection of mosquito cells we generated ZIKV variants for subsequent *in vitro* and *in vivo* studies.

### Recoded ZIKV Variants Show the Stability of *de novo* Introduced CpG Dinucleotides *in vitro* and *in vivo*

We assessed whether *de novo* introduced CpG dinucleotides in recoded ZIKV variants remain stable after multiple passages in cells and infection in animals. All ZIKV variants, except E/NS1+176CpG, showed infectious titers after 10 passages in VERO cells. The E/NS1+176CpG variant did not show infectious titers after the second passage; thus, the initial virus incubation step of 1 h was increased to 6 h in each passage. Before passaging on VERO cells, none of the ZIKV variants had mutations. After 10 *in vitro* passages, all *de novo* introduced CpG dinucleotides were preserved in all variants. The E+32CpG and E/NS1+176CpG variants had one nucleotide mutation in the genomic region encoding the E protein. In the E+32CpG variant the mutation U2405C was silent; while in the E/NS1+176CpG variant the mutation U2460G modified the protein sequence—L785V. Mutations were confirmed with repeated sequencing. In both ZIKV variants mutations did not affect the *de novo* introduced or original CpG content. *In vivo*, the E+32CpG RNA extracted from the mouse brain at 21 days after inoculation (see mouse neonatal experiment below) also showed silent U2405C mutation. All other recoded variants did not show mutations *in vivo*.

Altogether, *in vitro* and *in vivo* assays demonstrated the stability of *de novo* introduced CpG dinucleotides in all recoded ZIKV variants.

### ZIKV Variants With the Increased CpG Content Show Reduced Infection Kinetics *in vitro*

**Figure 3:**
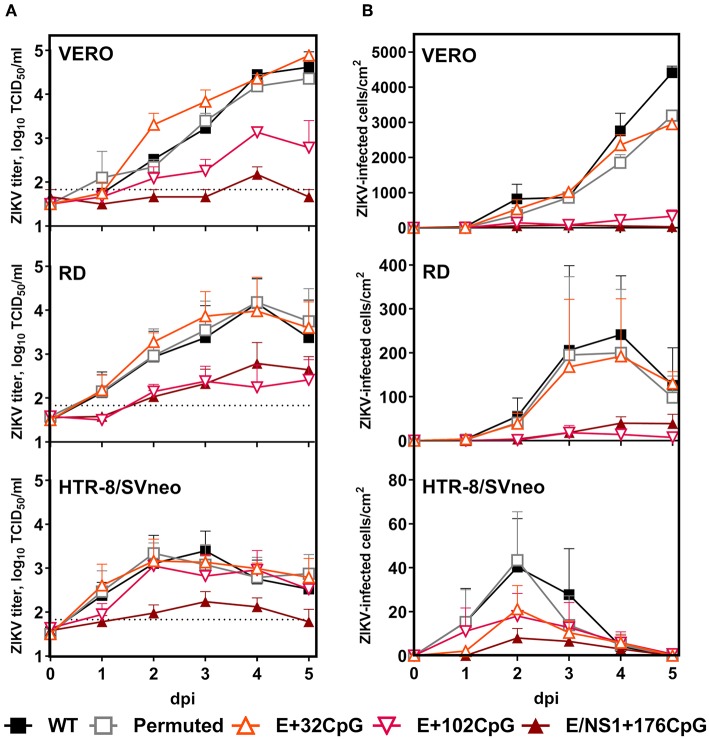
Virus infection kinetics in VERO, RD, and HTR-8/SVneo cells after inoculation at a MOI of 0.01. **(A)** Cell culture supernatants were collected and viral titers were measured using the endpoint dilution assay. The dotted horizontal line represents the limit of detection (LOD). **(B)** The 96-well plates with cell monolayers were stained with ZIKV-specific Abs and infected cells were counted in the whole well with bright-field microscopy at 200x (Figure 4). Whiskers represent standard error of the mean (SE) from three biologically independent replicates with three (two for virus titrations) technical replicates. “dpi:” days post inoculation. Dunnett's test showed multiplicity-adjusted statistically significant *p*-values for E+102CpG (*p* = 0.047, the number of ZIKV-infected RD cells) and E/NS1+176CpG (*p* = 0.014, ZIKV titers on HTR-8/SVneo; *p* = 0.019, ZIKV titers on VERO cells; and *p* = 0.020, ZIKV-infected VERO cells) vs. WT.

**Figure 4:**
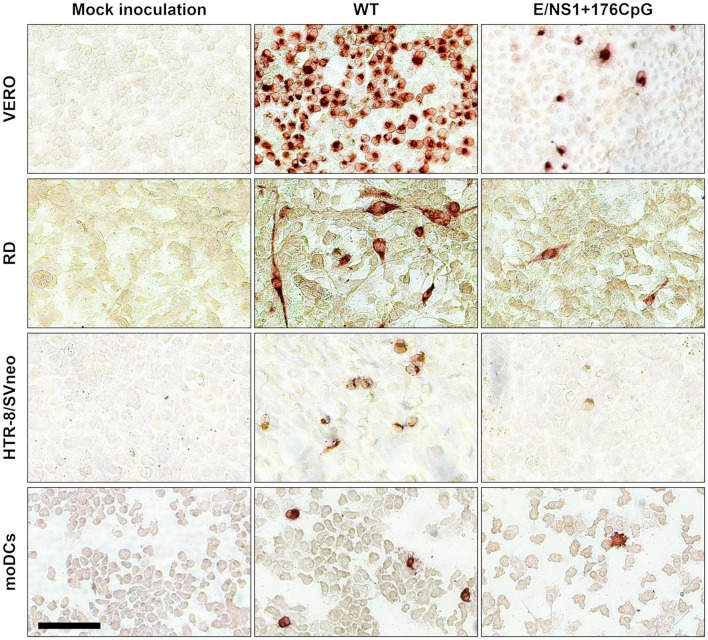
Representative ZIKV antigen staining in cell lines and human moDCs. Brown staining represents ZIKV-positive cells. The bar is 100 μm.

We used VERO, RD, and HTR-8/SVneo cell lines and human moDCs to compare infection kinetics of WT vs. CpG-recoded ZIKV variants. In all cell lines, WT, Permuted, and the E+32CpG variant—the one with the lowest CpG content among all recoded variants—had similar infection kinetics (Figure 3). In contrast, two other CpG-recoded variants showed distinct replication: In RD cells E+102CpG and NS1/E+176CpG recoded variants replicated more slowly producing lower infectious titers (Figure 3A) and a lower number of virus-positive cells (Figures 3B, 4). In VERO cells, replication of the E+102CpG and NS1/E+176CpG recoded variants was at or below the limit of detection (Figure 3). In both cell lines, peak viral titers for E+102CpG and NS1/E+176CpG variants were 1.5–1.7 and 1.4–2.4 log_10_ lower vs. the WT variant (Figure 3A). The same trend was confirmed by the quantification of virus-positive VERO and RD cells (Figures 3B, 4). In HTR-8/SVneo cells only the NS1/E+176CpG variant—the one with the highest CpG content among all recoded viruses—showed attenuation (Figure 3A).

**Figure 5:**
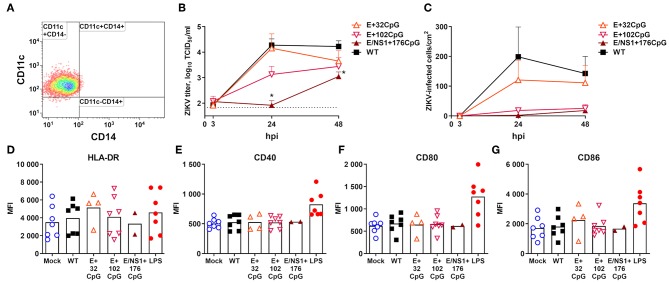
Virus infection kinetics in human moDCs after inoculation at MOI of 1. **(A)** MoDCs were generated from blood cells of eight adult females sampled at birth or shortly before birth and one adult male. Differentiation of CD14+ monocytes from human blood resulted in a typical moDC phenotype. In this experiment, we excluded the permuted ZIKV variant because the number of moDCs was limited. **(B)** An asterisk (*) denotes a statistically significant difference between WT and E/NS1+176CpG ZIKV variants. The dotted horizontal line represents the limit of detection. **(C)** The 96-well plates with moDC cells were stained with ZIKV-specific Abs and ZIKV-infected cells were counted in the well with bright-field microscopy at 200x (Figure 4). Whiskers represent SE from nine donors with two technical replicates. **(D–G)** Expression of flow cytometry markers in moDCs inoculated with ZIKV variants (MOI = 1) and measured at 48 h post inoculation. Bars represent mean values. LPS (100 ng/ml Lipopolysaccharide; Sigma L5418) was used as a positive control. MFI, mean fluorescent intensity; hpi, hours post inoculation.

In human moDCs (Figure 5A), the E+102CpG and NS1/E+176 CpG variants produced lower infectious titers (Figure 5B) and a lower number of virus-positive cells (Figures 4, 5C). In contrast, WT and E+32CpG variants produced comparable infectious titers and numbers of infected cells (Figures 5B,C). Infection with all ZIKV variants has not significantly affected the expression of moDC activation markers at 48 h after inoculation (Figures 5D–G).

In summary, increasing the ZIKV genomic CpG content reduced infection kinetics in mammalian cell lines and human moDCs; reduction consistently depended on the CpG content in recoded variants as vividly represented by similar infection kinetics of WT and E+32CpG variants.

### ZIKV Variants With the Increased CpG Content Show Reduced Fitness in *in vitro* Competition Assays

The competition assay, where the relative replicative fitness of two variants can be compared after co-infection and passage in cells, has been described as a sensitive tool to compare the fitness of CpG-recoded viruses (4, 5, 11). The E/NS1+176CpG variant was outcompeted by all other variants and fell to undetectable levels at passage 5 (Table 3). The E+102CpG variant was completely outcompeted by the E+32CpG, Permuted, and WT variants. In contrast, the E+32CpG variant—the one with the lowest CpG content among all recoded variants—was only partially outcompeted by the Permuted and WT ZIKV variants (Table 3). Wild-type and Permuted variants showed comparable replication fitness.

**Table 3:** *In vitro* competition assay with ZIKV variant-specific restriction digestion.

		**Competing mutant # 2**			
		**E+102CpG**	**E+32CpG**	**Permuted**	**Wild-type**
**Competing mutant #1^a^**	E/NS1+ 176CpG	–^b^ – –	– – –	– – –	– – –
	E+102CpG		– – –	– – –	– – –
	E+32CpG			– ≈ ≈	– ≈ ≈
	Permuted				≈ ≈ +

a *Relative fitness of the competing mutant #1 over the competing mutant #2 was evaluated in triplicate based on the digestion pattern (Supplementary Figure 1)*.

b *“–”: no competing mutant #1 after passaging; “+”: no competing mutant #2 after passaging; “≈”: both mutants present after passaging*.

Outcomes of the competition assay consistently depended on the genomic CpG content in ZIKV variants as represented by relative fitness ranking: WT ≈ Permuted ≥ E+32CpG > E+102CpG > E/NS1+176CpG.

### ZIKV Variants With the Increased CpG Content Cause Attenuated Infection Phenotypes in a Host-Age-Dependent Manner

We used a set of well-established mouse models (21–24, 40, 51–53) for ZIKV infection to determine how CpG recoding in the RNA virus genome affects infection phenotypes in hosts of different age—neonates and adults.

To compare infection phenotypes in neonates, we used the mouse model (24); mouse neonates are susceptible to infection with wild-type ZIKV, showing neurological clinical signs and lethality, and providing a sensitive experimental model to compare virus strains or live vaccine candidates (24, 51–53).

**Figure 6:**
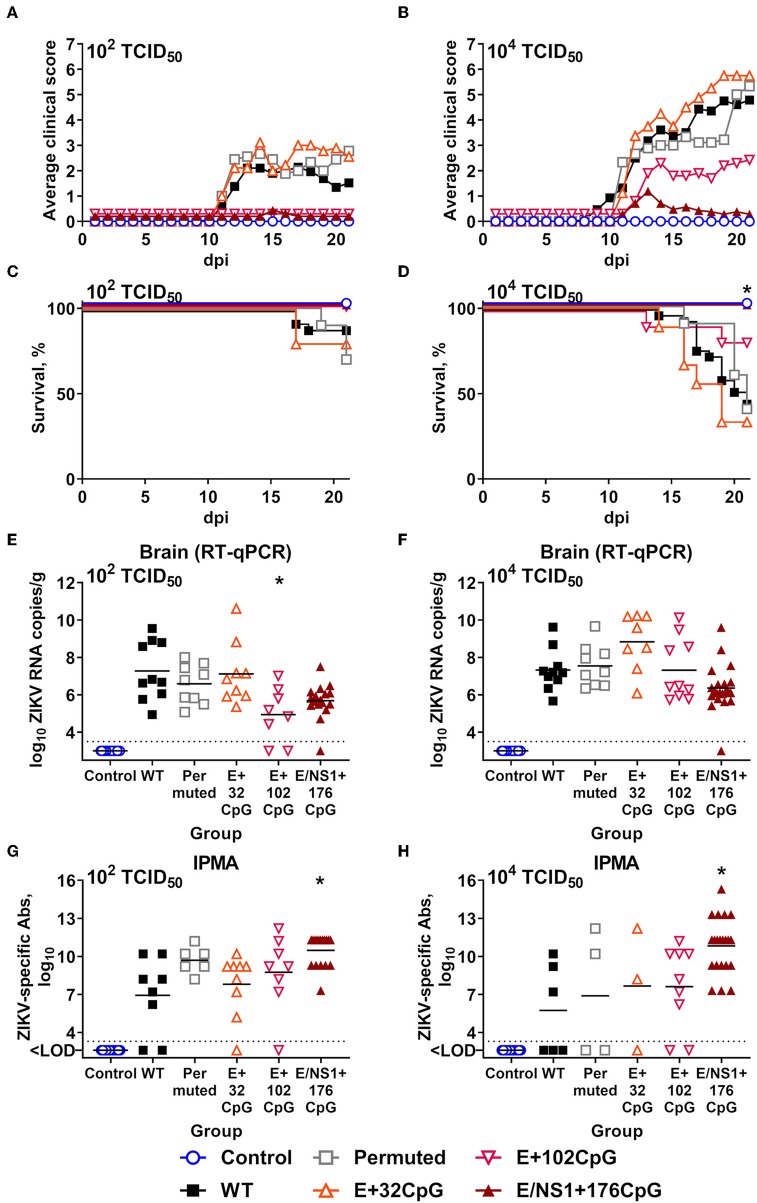
Infection phenotypes of ZIKV variants in neonatal mice. One-day-old pups (control: *n* = 19; ZIKV variants: *n* = 8–29) were inoculated with either 10^2^ or 10^4^ TCID_50_ of ZIKV. **(A,B)** Mean clinical scores: 0—no visible abnormalities; 1—mild ataxia and/or tremors; 2—obvious ataxia and/or tremors; 3—depression, hunching, reluctance to walk, and falling to the side when walking; 4—obvious hyperactivity; 5—close to moribund but still somewhat responsive; 6—paralysis; 7—death. **(C,D)** Percentage of survival. **(E,F)** ZIKV loads in brains sampled at 21 days after inoculation or death. **(G,H)** ZIKV-specific IgG Ab titers in blood plasma at 21 days after inoculation; quantified by immunoperoxidase monolayer assay—IPMA. Asterisk (*) denotes statistically significant difference compared to the WT ZIKV group. Dotted lines represent the LOD, solid lines represent mean values.

One-day-old immunocompetent mice were inoculated IP with 10^2^ or 10^4^ TCID_50_ of different ZIKV variants. Neonates infected with 10^2^ TCID_50_ of E+102CpG and NS1/E+176CpG recoded variants did not show clinical signs (Figure 6A); neonates infected with 10^4^ TCID_50_ of these variants showed mild to moderate clinical signs for E+102CpG and no clinical signs for NS1/E+176CpG (Figure 6B). Accordingly, neonates infected with 10^2^ TCID_50_ of E+102CpG and NS1/E+176CpG recoded variants did not show mortality (Figure 6C), while neonates infected with 10^4^ TCID_50_ of these variants showed 18% mortality for E+102CpG and no mortality for NS1/E+176CpG (Figure 6D). Mice infected with the WT, Permuted, or E+32CpG variant—the one with the lowest CpG content among all recoded viruses—showed similar severe clinical signs (Figures 6A,B) and high mortality (Figures 6C,D).

At 21 days post inoculation, mice infected with 10^2^ TCID_50_ of E+102CpG and NS1/E+176CpG variants had considerably lower (1.6–2.3 log_10_) mean viral loads in the brain than mice in other groups (Figure 6E). A statistically significant difference, however, was noted only for the E+102CpG group. Mice infected with 10^4^ TCID_50_ of WT or CpG-recoded variants had comparable viral loads in the brain (Figure 6F). Neonates infected with the E+32CpG variant had similar (10^2^ TCID_50_; Figure 6E) or 1.6 log_10_ higher (10^4^ TCID_50_, Figure 6F) viral loads in the brain in comparison to the WT variant. Mice infected with WT and Permuted variants had similar viral loads in brains (Figures 6E,F). Most animals in all groups developed ZIKV-specific Abs at 21 days after inoculation; only the NS1/E+176CpG group had significantly higher (*p* = 0.060–0.016) Ab titers than the WT group (Figures 6G,H).

To summarize, in comparison to the WT variant, recoded E+102CpG and NS1/E+176CpG ZIKV variants caused considerably reduced clinical disease and mortality in neonatal mice and a moderate dose-dependent reduction of viral loads.

To compare infection phenotypes in adult animals, we used the mouse pregnancy model (22, 40); maternal inoculation results in congenital infection and pregnancy pathology providing a sensitive experimental model to compare infection phenotypes caused by different strains or mutants. At E5, 8- to 10-weeks-old immunocompetent, bred dams were injected IP with 2 mg of anti-IFNAR1 Abs; at E6, mice were inoculated SC with 10^4^ TCID_50_ of WT, E+102CpG, or E/NS1+176CpG ZIKV variants or control virus-free media, and at E18, mice were sampled. Because in neonatal mice Permuted and E+32CpG ZIKV variants had an infection pattern similar to the WT variant (Figure 6), in this mouse experiment and subsequent mouse immunogenicity and challenge experiments only WT, E+102CpG, and E/NS1+176CpG variants were used.

**Figure 7:**
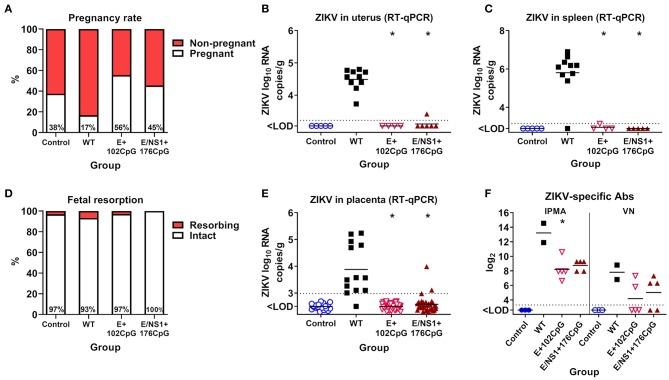
Infection phenotypes of CpG-recoded ZIKV variants in pregnant mice. **(A)** The pregnancy rate in mated and plugged mice (*n* = 8 dams, Control; *n* = 12, WT; *n* = 9, E+102CpG; *n* = 11, E/NS1+176CpG). ZIKV loads in the uterus **(B)** and spleen **(C)** of bred, non-pregnant mice. **(D)** The percentage of fetal resorption (*n* = 32 fetuses, Control; *n* = 15, WT; *n* = 35, E+102CpG; *n* = 33, E/NS1+176CpG). **(E)** ZIKV loads in the placenta. **(F)** Maternal ZIKV-specific IgG (quantified by immunoperoxidase monolayer assay—IPMA) and neutralizing (quantified by virus-neutralizing assay—VN) Abs in the pregnant subgroup. Asterisk (*) denotes a statistically significant difference compared to the WT ZIKV group. Dotted lines represent the LOD, solid lines represent mean values.

As expected, not all animals with vaginal plugs—the sign of mating and potential pregnancy in mice—had fetuses at sampling. Control mock-inoculated mice had a 38% pregnancy rate (Figure 7A); this is within the normal pregnancy rate of 31–44% documented by The Jackson Laboratory (USA) for healthy mice (54–56). The pregnancy rate in mice inoculated with recoded E+102CpG (56%) and NS1/E+176CpG (45%) variants was also within the expected rate and even higher than in the Control group (Figure 7A). In contrast, the pregnancy rate in mice inoculated with the WT variant was only 17% (Figure 7A). In accordance with these clinical findings, only mice in the non-pregnant WT ZIKV subgroup had viral loads in uterine and spleen samples (Figures 7B,C); maternal brain samples from the WT animals, however, were ZIKV-negative. Uterine, spleen, and maternal brain samples from the Control, E+102CpG, and NS1/E+CpG animals in the non-pregnant subgroup were negative for ZIKV (Figures 7B,C).

In the pregnant subgroup, fetal death was low (0–7%) in all ZIKV and Control groups (Figure 7D) and ZIKV was not detected in fetal heads. High ZIKV loads, however, were found in placental samples from only the WT group (Figure 7E); only three placental samples in the NS1/E+176CpG group had ZIKV loads at or just above the detection limit (Figure 7E). Maternal spleens and brains were ZIKV-negative. Pregnant dams in all groups developed ZIKV-specific and neutralizing Abs (Figure 7F).

Altogether, in comparison to the WT ZIKV variant, inoculation with recoded E+102CpG and NS1/E+176CpG variants did not reduce the pregnancy rate and did not cause overt infection in maternal internal organs and placenta.

### ZAP Expression in Animal Tissues Is Age-Dependent

**Figure 8:**
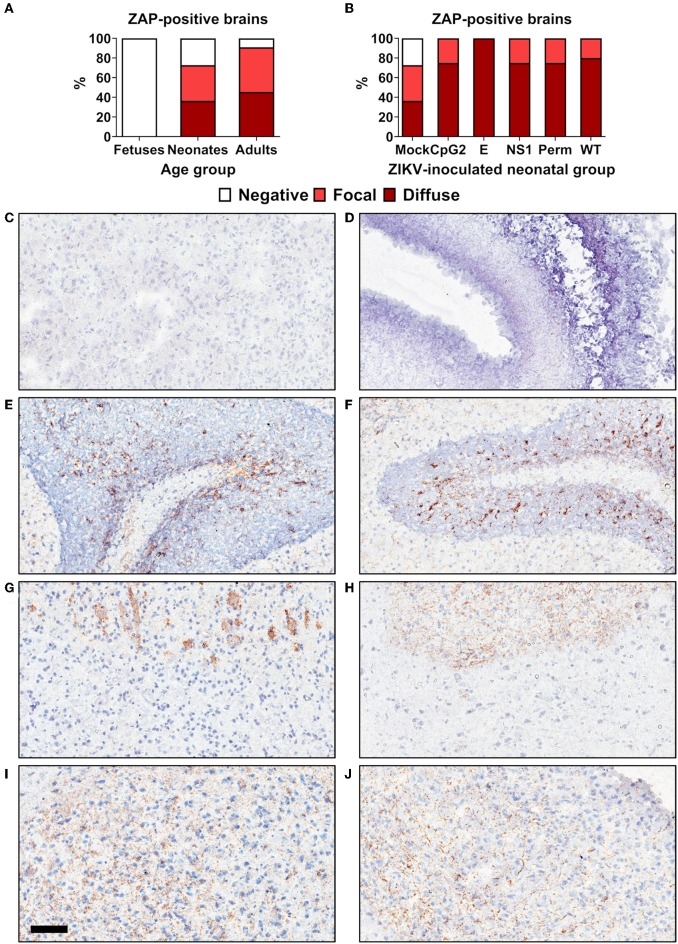
Age-dependent ZAP expression in brain tissues of mice. **(A)** ZAP expression in different age groups (*n* = 8–11 healthy animals). **(B)** ZAP expression in neonatal mouse groups inoculated with different ZIKV variants at 1 day and sampled at 21 days after birth (*n* = 4–11). **(C)** Negative staining control. **(D)** Fetal brain. **(E)** Neonatal cerebellum. **(F)** Adult cerebellum. Focal staining in neonatal **(G)** and adult **(H)** white matter. Diffuse staining in neonatal **(I)** and adult **(J)** white matter. The scale bar is 100 μm.

We further compared ZAP expression in mouse brains from fetuses, neonates, and adults because ZAP is the major known immune component targeting viral CpG dinucleotides (9, 14). Immunohistochemistry did not reveal ZAP-positive cells in non-infected fetal brains (Figures 8A,D). In contrast, ZAP expression was uniformly found in the neonatal and adult cerebellum from healthy animals (Figures 8E,F). In both age groups, the pattern of ZAP staining in the white matter was heterogeneous—ranging from negative, to focal, to diffuse (Figures 8A,G–J). And the intensity of staining also varied from low to high. Brains from healthy adults showed a trend to higher ZAP expression in comparison to brains from healthy neonates (Figure 8A); however, a statistically significant difference was not determined (*p* = 0.58). Neonatal brain infection with all ZIKV variants shifted the ZAP expression toward more uniform (diffuse) staining across all white matter (Figure 8B).

In support of the host-age-dependent attenuation of infection phenotypes caused by CpG-recoded viruses we demonstrated that expression of ZAP—the host protein targeting viral CpG dinucleotides—is also age-dependent.

### ZIKV Variants With the Increased CpG Content Induce Cellular Immune Responses in Adult Mice

**Figure 9:**
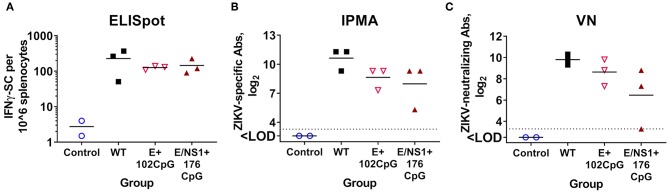
Cellular **(A)** and humoral **(B,C)** immune responses in adult mice 28 days after immunization with WT and CpG-recoded ZIKV variants. ELISpot, enzyme-linked immune absorbent spot; IPMA, immunoperoxidase monolayer assay; VN, virus-neutralizing assay. Dotted lines represent the LOD, solid lines represent mean values.

After infection with WT, E+102CpG and NS1/E+176CpG variants, neonatal (Figure 6) and adult mice (Figure 7) consistently developed humoral responses with high ZIKV-specific Ab titers. Here, we tested whether immunization with CpG-recoded ZIKV variants results in cell-mediated immune responses in adult mice. All mice immunized with the WT, E+102CpG and NS1/E+176CpG variants showed a 46–82-fold increase in the number of IFN-γ-secreting cells in comparison to non-immunized control mice (Figure 9A). Immunized animals also developed comparable ZIKV-specific and neutralizing Abs (Figures 9B,C).

Despite attenuated infection in adults (Figure 7) immunization with CpG-recoded E+102CpG and NS1/E+176CpG variants induced cellular and humoral immune responses comparable to responses induced by the WT variant.

### Immunization With CpG-Recoded ZIKV Variants Protects Mice Against Heterologous Lethal Challenge

**Figure 10:**
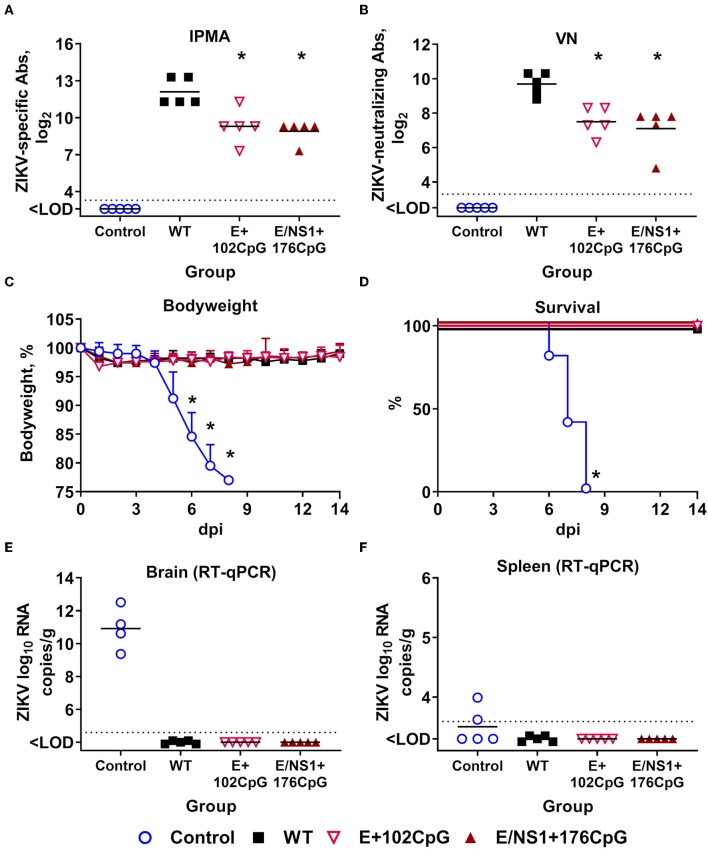
Protective efficacy of WT and CpG-recoded ZIKV variants in adult mice. ZIKV-specific **(A)** and neutralizing **(B)** Abs at 28 days after immunization with 10^5^ TCID_50_ of WT or CpG-recoded ZIKV variants. IPMA, immunoperoxidase monolayer assay; VN, virus-neutralizing assay. Bodyweight **(C**; bodyweight at day 0 was used as a baseline**)**, survival **(D)**, and ZIKV loads in the brain **(E)** and spleen **(F)** after IC challenge with 10^5^ TCID_50_ of the heterologous mouse-adapted ZIKV MR-766 strain. Asterisk (*) denotes a statistically significant difference to the WT ZIKV group. Dotted lines represent the LOD **(A,B,E,F)**; solid lines represent mean values.

**Figure 11:**
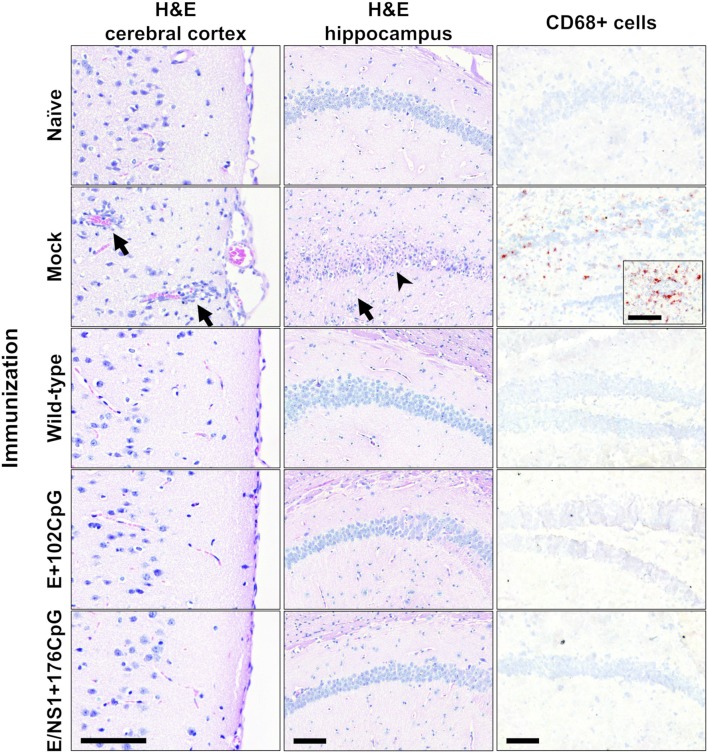
Histopathology and immunohistochemistry in mouse brains. H&E: Cortical monocyte infiltration, inflamed cortical blood vessels (arrows), hippocampal monocyte invasion (arrow), and necrosis of hippocampal neurons (arrowhead) were detected only in mock-immunized mice. Immunohistochemistry: CD68-positive cells were detected in brains from only mock-immunized mice. Brains from all control and experimental animals (Figure 10) were tested. Scale bars are 100 μm.

Using the established model (23), we further assessed whether immunity induced by WT and CpG-recoded ZIKV variants evokes comparable protective efficacy against lethal challenge with the heterologous mouse-adapted ZIKV MR-766 strain. Immunization of adult mice with all ZIKV variants resulted in robust ZIKV-specific and neutralizing Ab responses (Figures 10A,B). None of the immunized animals exhibited clinical disease (Figures 10C,D). In contrast, mock-immunized animals lost weight and died abruptly (Figures 10C,D). Only mock-immunized animals had high viral loads in brains (Figure 10E) and two out of five spleen samples from mock-immunized mice also had ZIKV loads (Figure 10F). Accordingly, only mock-immunized mice had brain histopathology represented by monocyte infiltration around brain blood vessels and apparent neuronal necrosis and monocyte invasion in the hippocampus (Figure 11). These histopathological findings (H&E staining) were consistent with previously described histopathology in ZIKV-infected mouse brains (45, 46). Supporting histopathological findings, immunohistochemistry demonstrated CD68-positive cells—a lysosomal protein that is highly expressed in activated microglia (57)—in the brain from only mock-vaccinated animals (Figure 11); CD68-positive cells were focally located in the white matter, cortex, around blood vessels, and diffusely in the hippocampus.

In summary, immunization of adult animals with WT or CpG-recode variants evoked high protective efficacy against lethal heterologous challenge with mouse-adapted ZIKV.

## Discussion

Increasing the number of CpG dinucleotides in viral genomes is a promising vaccine approach; attenuated infection phenotypes caused by recoded vaccine candidates, however, may depend on the expression of cellular components targeting viral CpG dinucleotides (7, 15). Thus, population-based studies on infection caused by CpG-recoded viruses will foster further application of the approach. To our knowledge, side-by-side comparative studies in different age groups have not been reported for CpG-recoded viruses. In the present study, we generated several ZIKV variants with the gradually increasing CpG content and compared infection phenotypes in different age groups. We used ZIKV as a model because it causes infection in hosts of different ages—neonates and adults. The protective efficacy of immunization against lethal challenge was also tested. We found that increasing the CpG content in the ZIKV virus genome causes host-age-dependent attenuation of infection phenotypes with considerable attenuation of clinical disease and moderate reduction of viral loads in neonates and high attenuation in adults. In support of the host-age-dependent attenuation of infection phenotypes we demonstrated that expression of ZAP—the host protein targeting viral CpG dinucleotides—is also age-dependent. Similar to the WT variant, ZIKV variants with the increased CpG content evoked robust cellular and humoral immune responses and protection against lethal challenge in adult animals. Altogether, the *in vivo* experimental findings presented here may contribute to a more detailed understanding of age-dependent host mechanisms targeting viral CpG dinucleotides and potential practical applications of CpG-recoded vaccines in the future.

### Host-Age-Dependent Attenuation of Infection Phenotypes Caused by CpG-Recoded Viruses

We generated ZIKV variants using general principles formulated for CpG recoding (5); this resulted in variants with the gradually increasing CpG content (Figure 2), unaltered encoding proteins, similar ENc and CAI values vs. WT sequences (Table 2), and minimal changes in CPB values at the ORF level (Table 2). In contrast to the E+32CpG variant—the one with the lowest CpG content among all recoded variants—the E+102CpG and NS1/E+176CpG variants showed the expected reduced replication in VERO and RD cells lines (Figure 3). This dissonance in replication kinetics of different CpG-recoded variants and consistent results of the competition assay with relative fitness ranking—WT ≈ Permuted ≥ E+32CpG > E+102CpG > E/NS1+176CpG (Table 3), demonstrate that *in vitro* ZIKV replication can be tuned by adjusting the number of *de novo* introduced CpG dinucleotides.

Only CpG-recoded influenza virus has been extensively studied in adult mouse models (4). Codon pair deoptimized viruses were studied in animal models as well (58, 59); however, effects of codon pair bias deoptimization may arise through the unintended increase in CpG and UpA dinucleotide frequencies (6, 7, 9, 11–13, 60). For example, in a recent ZIKV study (61), codon pair bias deoptimization was associated with a considerable increase in both CpG (294–455%) and UpA (185–371%) dinucleotides. Thus, *in vivo* studies with CpG-recoded viruses, not biased by UpA recoding and codon pair bias deoptimization, remain limited. Here, we expanded *in vivo* infection studies to different age groups, specifically to neonatal and adult periods, and demonstrated that increasing the CpG content in the RNA virus genome causes the host-age-dependent attenuation of infection phenotypes.

Infection with CpG-recoded E+102CpG and NS1/E+176CpG variants in neonates resulted in reduced clinical disease and mortality as compared to WT ZIKV. The E+32CpG variant—the one with the lowest CpG content among recoded variants—showed similar or exacerbated clinical signs and mortality, and similar or higher viral loads in brains (Figure 6) compared to WT ZIKV. This virus CpG content-dependent pattern demonstrates that similar to *in vitro* infection kinetics, *in vivo* infection can be modulated by adjusting the number of *de novo* introduced CpG dinucleotides. We do not know the mechanisms of milder clinical disease and mortality caused by E+102CpG and NS1/E+176CpG variants because both recoded variants crossed the neonatal blood-brain barrier, showing only moderately reduced or similar viral loads in the brain as compared to WT ZIKV. A recently raised concern suggests that CpG-recoded neurotropic viruses may show restricted replication at sites of entry but may lose attenuation once they enter the central nervous system (14); this concern was raised because *in silico* analysis showed that expression of ZAP was lower in the central nervous system in comparison to other internal organs. One limitation of our study is that only brains were available for comparative ZAP analysis (Figure 8).

Further supporting the host-age-dependent attenuation of infection, inoculation with CpG-recoded variants in adult pregnant mice did not cause a reduction in the pregnancy rate or detectable virus replication in maternal organs. In contrast, the WT ZIKV variant considerably reduced the pregnancy rate and efficiently replicated in maternal spleen and uterus (Figure 7). Critically, the absence of E+102CpG infection and only minimal infection caused by NS1/E+176CpG in the highly susceptible placenta suggest that variants with the increased CpG content are very efficiently targeted and attenuated by the immunity of the adult host. It is not clear, however, why the NS1/E+176CpG variant—the one with the highest CpG content—showed detectable viral loads in several uterine and placenta samples (Figures 7B,E); as suggested (17), most probably each vaccine candidate would require rigorous testing of a pathogen-specific CpG-recoding approach.

It has been recently demonstrated that ZAP is the major host immune component targeting viral CpG dinucleotides (9, 14); thus, attenuated infection caused by recoded viruses may depend on host- or population group-specific expression of ZAP. In contrast to neonatal and adult brains, we did not identify ZAP expression in fetal brains (Figures 8A,D). Brains from healthy adults showed a trend toward higher ZAP expression in comparison to brains from healthy neonates; however, a statistically significant difference was not determined (*p* = 0.58). This age-specific pattern of ZAP expression supports and provides a mechanistic background for the observed host-age-dependent attenuation of infection phenotypes caused by viruses with the increased CpG content.

### Immunogenicity and Protective Efficacy of CpG-Recoded ZIKV Variants

Recoded vaccine candidates with the increased CpG content encode all viral proteins with the same amino acid sequence as the wild-type prototype potentially inducing similar immune responses (17). In support of this, immunization with CpG-recoded ZIKV variants evoked robust cellular and humoral immune responses comparable to WT ZIKV (Figure 9), and protection against lethal challenge with the heterologous mouse-adapted ZIKV strain (Figure 10). In DNA molecules, CpG dinucleotides can directly activate B cells, natural killer cells, DCs, monocytes, and macrophages through TLR9 stimulation (62); introduced CpG dinucleotides in synthetic RNA molecules also may activate cellular immune responses, however, the mechanisms of activation remain unclear (63). Whether the increased CpG content in recoded RNA viruses leads to more efficient immune cell activation and augmented protective efficacy remains to be determined. To partially address this question, we studied infection phenotypes and immune activation caused by ZIKV variants in human moDCs. Dendritic cells are critical cells which bridge virus detection to activation of antiviral host immunity during natural infection or vaccination. Many viruses, however, have evolved to overcome DC responses; for example, ZIKV infects human DCs and does not cause their activation (64–66). We explored such ZIKV-DC interactions as a model to define whether increasing of CpG dinucleotides in the RNA viral genome may serve as an approach to subvert impaired antiviral responses in DCs. The CpG-recoded E+102CpG and E+176CpG ZIKV variants caused impaired infection in moDCs as compared to the WT and E+32CpG variants (Figure 5); however, in accordance with previous studies with wild-type ZIKV (64–66), activation of moDCs was not induced by any ZIKV variant as represented by similar expression of HLA-DR, CD40, CD80, and CD86 (Figures 5D–G). Despite attenuated infection caused by recoded variants and no overt immune activation, infected DCs may potentially migrate to draining lymph nodes and present proteins of recoded viruses to T cells with the same efficiency as WT ZIKV—in support, mice immunized with WT and recoded variants showed a similar level of ZIKV-specific humoral and cellular responses (Figure 9). If confirmed *in vivo*, the attenuated infection phenotypes in DCs caused by recoded vaccine candidates could prove to be a useful property for CpG-recoded vaccines because DC-mediated antigen presentation can occur efficiently without or with less further virus amplification in lymph nodes. Also, to better understand interactions between the increased CpG content in RNA viral genomes and DCs, future studies should focus on infection caused by other viruses which, in contrast to ZIKV, do not suppress immune activation in DCs.

### Implications for CpG Recoding Vaccine Approach

Data available from CpG-recoded influenza (4) and the present *in vivo* studies with ZIKV encourage further evaluation of recoded vaccine candidates in mammalian and particularly non-human primate models, and potentially in clinical trials. Experience in the vaccine development field suggests that modified live vaccines, in general, produce more robust immunity, including longer duration of immunity, compared to other vaccine constructs (67). However, the same safety concerns as for LAVs—population-based side effects, potential reversion to virulence and transmissibility—could also be attributed to CpG-recoded vaccines. The host-age-dependent pattern of infection phenotypes suggests that CpG-recoded vaccines may be safe in a healthy adult population before pregnancy, providing rapid and potentially long-term protection, which is particularly important during unfolding outbreaks.

Reversion to virulence is another safety concern. Recoded vaccine candidates with hundreds extra CpG dinucleotides most probably will show rare or no reversion to virulence; the stability of *de novo* introduced CpGs during *in vitro* and *in vivo* ZIKV infection in our current study further supports this assumption. The introduction of deattenuating mutations within modified, adjacent, or distant genomic regions which do not affect CpG dinucleotides may also be an issue, particularly under strong selection pressure (17). Finally, safety concerns due to persistent infection caused by CpG-recoded vaccines, as exemplified by ZIKV persistence in neonates, and potential for individual-to-individual and vector-borne transmission will need to be addressed.

One of the potential perspectives for the CpG recoding vaccine approach is in combination with established LAV strategies; for example, CpG recoding can serve as an additional safety level for viruses bearing attenuating amino acid mutations. Often, virus attenuation in efficient LAVs is determined by single or several amino acid mutations—e.g., a systemic approach to the development of interferon-restricted ZIKV variants with properties similar to yellow fever 17D LAV resulted in a promising vaccine candidate with only several amino acid mutations (40). Another example is poliovirus vaccine Sabin strains with only a single mutation critical for attenuation (18). This reliance on a small number of critical mutations in vaccine strains may lead to reversion to virulence, recombination events, and depletion of the natural CpG content (68). Rationally increasing the CpG dinucleotide content in attenuated vaccine strains containing a small number of critical attenuating amino acid mutations may reduce the potential for reversion to virulence and vaccine-derived outbreaks.

Collectively, our results show host-age-dependent attenuation of ZIKV variants with the increased CpG content in a mammalian host. We also demonstrated high protective efficacy of immunization with CpG-recoded variants against lethal challenge in adult animals. These findings should encourage further efforts to understand age-dependent mechanisms of CpG-mediated virus attenuation, fine-tuning of the CpG-recoding technology for better vaccine safety and efficacy, and rigorous testing of recoded viruses in different biological groups under various physiological conditions.

## Data Availability Statement

All datasets generated for this study are included in the article/Supplementary Material.

## Ethics Statement

The study was carried out in accordance with the recommendations of the University of Saskatchewan Policy for Research Involving Human Participants. The protocol (#16-135) was approved by the University of Saskatchewan Biomedical Research Ethics Board. All human subjects were adults and provided written informed consent. Animal experiments were performed following the Canadian Council on Animal Care guidelines for humane animal use and were approved by the University of Saskatchewan's Animal Research Ethics Board (#20180012). All efforts were made to minimize animal suffering. Mice were euthanized by deeply anesthetizing them with isoflurane in a chamber, followed by exsanguination via cardiac puncture. To ensure death, the chest cavity was opened during tissue collection.

## Author Contributions

Conceptualization, project administration, and supervision: UK. Data curation: IT, DU, CW, and UK. Formal analysis and validation: IT, DU, and UK. Funding acquisition: IT, M-JM, VG, and UK. Investigation: IT, DU, NB, CW, and UK. Methodology, software, visualization, and writing—original draft preparation: IT and UK. Resources: CW, M-JM, VG, and UK. Writing—review and editing: IT, DU, NB, CW, M-JM, VG, and UK.

### Conflict of Interest

The authors declare that the research was conducted in the absence of any commercial or financial relationships that could be construed as a potential conflict of interest.
