# Supplementary material for: CpG-Recoding in Zika Virus Genome Causes Host-Age-Dependent Attenuation of Infection With Protection Against Lethal Heterologous Challenge in Mice
Source: Front Immunol. 2020 Jan 24;10:3077. doi: 10.3389/fimmu.2019.03077 (PMC6993062; doi:10.3389/fimmu.2019.03077)
Supplement: Supplementary file 1 [file Data_Sheet_1.PDF]

Supplementary Material

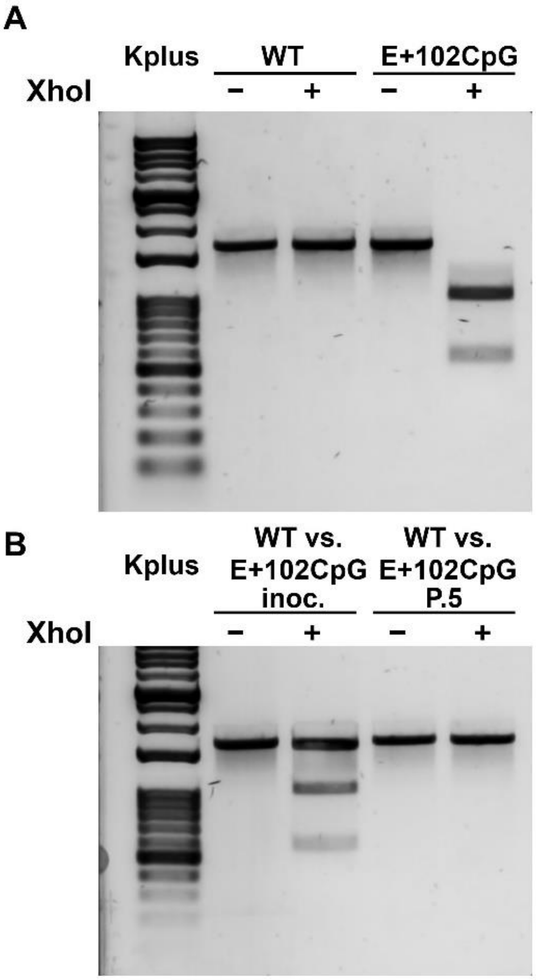

Supplementary Figure 1. An example of the competition assay of dual infection with WT and E+102CpG ZIKV variants. **(A)** Individual *XhoI* digestion profiles for WT and E+102CpG ZIKV variants. **(B)** *XhoI* digestion profiles in the WT versus E+102CpG ZIKV competition assay. The relative fitness ranking was WT > E+102CpG. "inoc."—initial inoculum; "P.5"—5th passage.

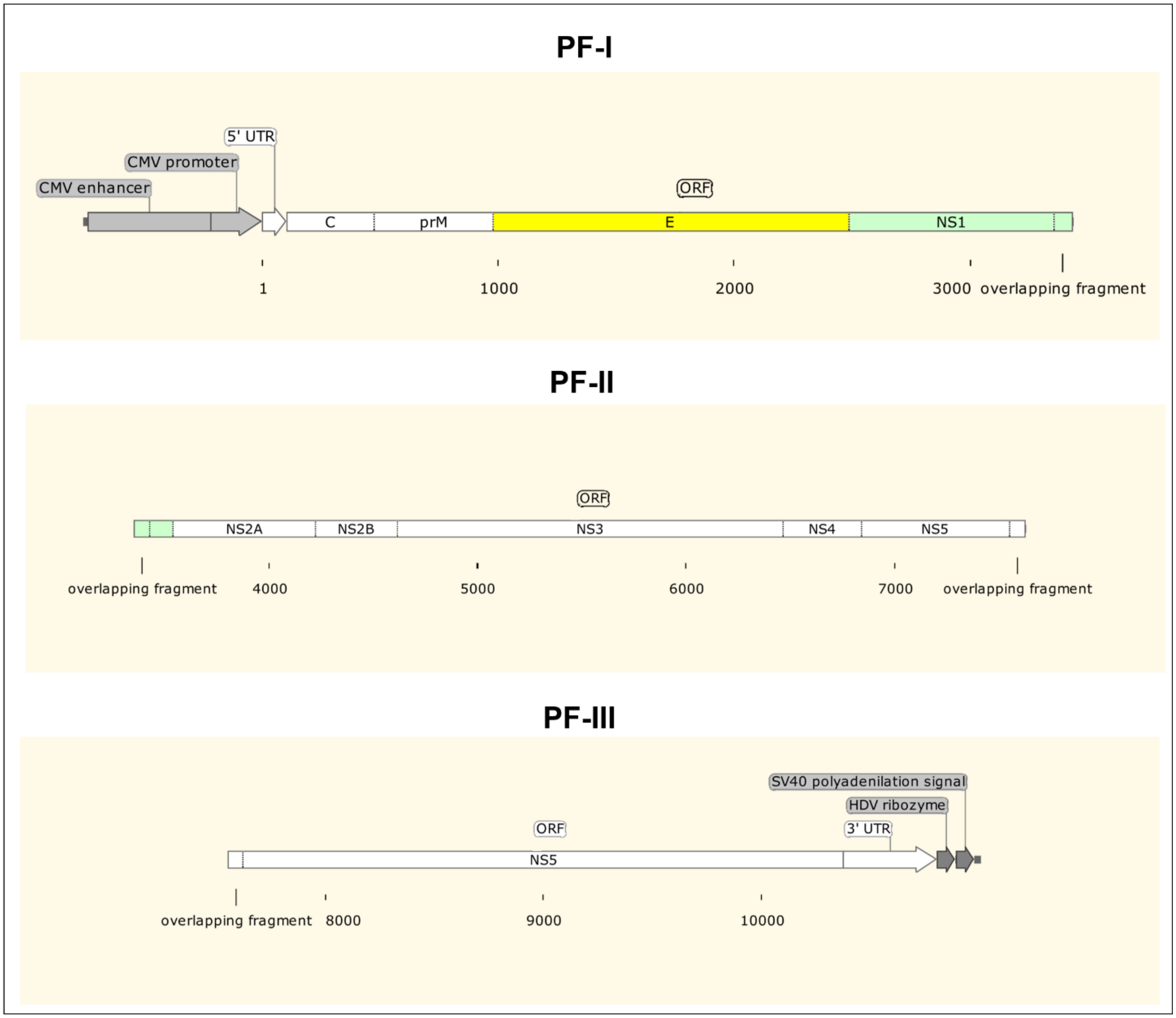

Supplementary Figure 2. A schematic map of PF-I (1-3428 nt), PF-II (3354-7621 nt), and PF-III (7553-10807 nt) fragments [Atieh T, *et al.* Simple reverse genetics systems for Asian and African Zika viruses. *Sci Rep* (2016) **6**:39384. doi:10.1038/srep39384]. Genomic regions encoding E and NS1 proteins (highlighted in yellow and green) were recoded using the MUTATE SEQUENCES program in the SSE 1.3 software package. All positions are based on complete genome sequence of the ZIKV H/PF/2013 strain [GenBank: KJ776791.2].

>KJ776791.2_Zika_virus_strain_H/PF/2013_WT

agttgttgatctgtgtgaatcagactgcgacagttcgagtttgaagcgaaagctagcaacagtatcaacaggttttattttggatttggaaacgagagtttctggtcatgaaaaacccaaaaaagaaatccggag
gattccggattgtcaatatgctaaaacgcggagtagcccgtgtgagcccctttgggggcttgaagaggctgccagccggacttctgctgggtcatgggcccatcaggatggtcttggcgattctagcctttttga
gattcacggcaatcaagccatcactgggtctcatcaatagatggggttcagtggggaaaaaagaggctatggaaataataaagaagttcaagaaagatctggctgccatgctgagaataatcaatgctaggaagg
agaagaagagacgaggcgcagatactagtgtcggaattgttggcctcctgctgaccacagctatggcagcggaggtcactagacgtgggagtgcatactatatgtacttggacagaaacgacgctggggaggcca
tatcttttccaaccacattggggatgaataagtgttatatacagatcatggatcttggacacatgtgtgatgccaccatgagctatgaatgccctatgctggatgaggggggtggaaccagatgacgtcgattgtt
ggtgcaacacgacgtcaacttgggttgtgtacggaacctgccatcacaaaaaaggtgaagcacggagatctagaagagctgtgacgctcccctcccattccactaggaagctgcaaacgcggtcgcaaacctggt
tggaatcaagagaatacacaaagcacttgattagagtcgaaaattggatattcaggaaccctggcttcgcgttagcagcagctgccatcgcttggcttttgggaagctcaacgagccaaaaagtcatatacttgg
tcatgatactgctgattgccccggcatacagcatcaggtgcataggagtcagcaatagggactttgtggaaggtatgtcaggtgggacttgggttgatgttgtcttggaacatggaggttgtgtcac**CG**taatgg
cacaggacaaac**CG**actgt**CG**acatagagctggttacaacaacagtcagcaacatgg**CG**gaggtaagatcctactgctatgaggcatcaatat**CG**gacatggctt**CG**gacagc**CG**ctgcccaacacaaggtgaag
cctaccttgacaagcaatcagacactcaatatgtctgcaaaagaa**CG**ttagtggacagaggctggggaaatggatgtggactttttggcaaagggagcctggtgacatg**CG**ctaagtttgcatgctccaagaaaa
tgac**CG**ggaagagcatccagccagagaatctggagtac**CG**gataatgctgtcagttcatggctcccagcacagtgggatgat**CG**ttaatgacacaggacatgaaactgatgagaatagag**CG**aaggttgagataa
CGcccaattcaccaagagc**CG**aagccaccctggggggttttggaagcctaggacttgattgtgaac**CG**aggacaggccttgacttttcagatttgtattacttgactatgaataacaagcactggttggttcaca
aggagtggttcca**CG**acattccattaccttggca**CG**ctggggcagacac**CG**gaactccacactggaacaacaaagaagcactggtagagttcaagga**CG**cacatgccaaaaggcaaactgt**CG**tggttctaggga **E**
gtcaagaaggagcagttcaca**CG**gcccttgctggagctctggaggctgagatggatggtgcaaagggaaggctgtcctctggccacttgaaatgt**CG**cctgaaaatggataaacttagattgaaggg**CG**tgtcat
actccttgtgtac**CG**cag**CG**ttcacattcaccaagatcc**CG**gctgaaacactgca**CG**ggacagtcacagtggaggtacagta**CG**cagggacagatggaccttgcaaggttccagctcagatgg**CG**gtggacatgc
aaactctgaccccagttgggaggttgataac**CG**ctaaccc**CG**taatcactgaaagcactgagaactctaagatgatgctggaacttgatccaccatttggggactcttacattgtcataggagt**CG**gggagaaga
agatcacccaccactggcacaggagtggcagcaccattggaaaagcatttgaagccactgtgagaggtgccaagagaatggcagtcttgggagacacagcctgggactttggatcagttggagg**CG**ctctcaact
cattgggcaagggcatccatcaaattttggagcagctttcaaatcattgtttggaggaatgtcctggttctcacaaattctcattggaa**CG**ttgctgatgtggttgggtctgaacacaaagaatggatctattt
cccttatgtgcttggccttaggggagtgttgatcttcttatccacagctgtctctgctgatgtggggtgct**CG**gtggacttctcaaagaaggaga**CG**agatg**CG**gtacaggggtgtt**CG**tctataa**CG**a**CG**ttg
aagcctggagggacaggtacaagtaccatcctgactcccc**CG**tagattggcagcagcagtcaagcaagcctgggaagatggtatctgtgggatctcctctgtttcaagaatggaaaacatcatgtggagatcag
tagaaggggagctcaa**CG**caatcctggaagagaatggagttcaactga**CG**gt**CG**ttgtgggatctgtaaaaaaccccatgtggagaggtccacagagattgcc**CG**tgcctgtgaa**CG**agctgcccca**CG**gctgga
aggcttgggggaaat**CG**tactt**CG**tcagagcagcaaagacaaataacagctttgt**CG**tggatggtgacacactgaaggaatgcccactcaaacatagagcatggaacagctttcttgtggaggatcatgggtt**CG** **NS1**
gggtatttcacactagtgtctggctcaaggttagagaagattattcattagagtgtgatccagc**CG**ttattggaacagctgttaagggaaaggaggctgtacacagtgatctaggctactggattgagagtgaga
agaatgacacatggaggctgaagagggcccatctgat**CG**agatgaaaacatgtgaatggccaaagtcccacacattgtggacagatggaatagaagagagtgatctgatcatacccaagtctttagctgggccac
tcagccatcacaataccagagagggctacaggacccaaatgaaagggccatggcacagtgaagagcttgaaatt**CG**gtttgaggaatgcccaggcactaaggtcca**CG**tggaggaaacatgtggaacaagaggac
catctctgagatcaaccactgcaag**CG**gaagggtgat**CG**aggaatggtgctgcagggagtgcacaatgcccccactgt**CG**ttc**CG**ggctaaagatggctgttggtatggaatggagataaggcccaggaaagaac
cagaaagtaacttagtaaggtcaatggtgactgcaggatcaactgatcacatggatcacttctcccttggagtgcttgtgattctgctcatggtgcaggaaggctgaagaagagaatgaccacaaagatcatca
taagcacatcgatggcagtgctggtagctatgatcctgggaggattttcaatgagtgacctggctaagcttgcaattttgatgggtgccaccttcgcggaaatgaacactggaggagatgtagctcatctggcgc
tgatagcggcattcaaagtcagaccagcgttgctggtatctttcatcttcagagctaattggacaccccgtgaaagcatgctgctggccttggcctcgtgtcttttgcaaactgcgatctccgccttggaaggcg
acctgatggttctcatcaatggttttgctttggcctggttggcaatacgagcgatggttgttccacgcactgataacatcaccttggcaatcctggctgctctgacaccactggcccggggcacactgcttgtgg
cgtggagagcaggccttgctacttgcgggggggtttatgctcctctctctgaagggaaaaggcagtgtgaagaagaacttaccatttgtcatggccctgggactaaccgctgtgaggctggtcgaccccatcaacg
tggtgggactgctgttgctcacaaggagtgggaagcggagctggcccccctagcgaagtactcacagctgttggcctgatatgcgcattggctggagggttcgccaaggcagatatagagatggctgggcccatgg
ccgcggtcggtctgctaattgtcagttacgtggtctcaggaaagagtgtggacatgtacattgaaagagcaggtgacatcacatgggaaaaagatgcggaagtcactggaaacagtccccggctcgatgtggcgc
tagatgagagtggtgatttctccctggtggaggatgacggtcccccatgagagagatcatactcaaggtggtcctgatgaccatctgtggcatgaacccaatagccatacccttttgcagctggagcgtggtacg
tatacgtgaagactggaaaaaggagtggtgctctatgggatgtgcctgctcccaaggaagtaaaaaagggggagaccacagatggagtgtacagagtaatgactcgtagactgctaggttcaacacaagttggag
tgggagttatgcaagaggggggtctttcacactatgtggcacgtcacaaaaggatccgcgctgagaagcggtgaagggagacttgatccatactggggagatgtcaagcaggatctggtgtcatactgtggtccat
ggaagctagatgccgcctgggacgggcacagcgaggtgcagctcttggccgtgccccccggagagagagcgaggaacatccagactctgcccggaatatttaagacaaaggatggggacattggagcggttgcgc
tggattacccagcaggaacttcaggatctccaatcctagacaagtgtgggagagtgataggactttatggcaatggggtcgtgatcaaaaatgggagttatgttagtgccatcacccaagggaggagggaggaag
agactcctgttgagtgcttcgagccttcgatgctgaagaagaagcagctaactgtcttagacttgcatcctggagctgggaaaaccaggagagttcttcctgaaatagtccgtgaagccataaaaacaagactcc
gtactgtgatcttagctccaaccagggttgtcgctgctgaaatggaggaagcccttagagggcttccagtgcgttatatgacaacagcagtcaatgtcacccactctggaacagaaatcgtcgacttaatgtgcc
atgccaccttcacttcacgtctactacagccaatcagagtccccaactataatctgtatattatggatgaggcccacttcacagatccctcaagtatagcagcaagaggatacatttcaacaagggttgagatgg
gcgaggcggctgccatcttcatgaccgccacgccaccaggaacccgtgacgcatttccggactccaactcaccaattatggacaccgaagtggaagtcccagagagagcctggagctcaggctttgattgggtga
cggatcattctggaaaaacagtttggtttgttccaagcgtgaggaacggcaatgagatcgcagcttgtctgacaaaggctggaaaacgggtcatacagctcagcagaaagacttttgagacagagttccagaaaa
caaaacatcaagagtgggactttgtcgtgacaactgacatttcagagatgggcgccaactttaaagctgaccgtgtcatagattccaggagatgcctaaagccggtcatacttgatggcgagagagtcattctgg
ctggacccatgcctgtcacacatgccagcgctgcccagaggagggggcgcataggcaggaatcccaacaaacctggagatgagtatctgtatggaggtgggtgcgcagagactgacgaagaccatgcacactggc
ttgaagcaagaatgctccttgacaatatttacctccaagatggcctcatagcctcgctctatcgacctgaggccgacaaagtagcagccattgagggagagttcaagcttaggacggagcaaaggaagacctttg
tggaactcatgaaaagaggagatcttcctgtttggctggcctatcaggttgcatctgccggaataacctacacagatagaagatggtgctttgatggcacgaccaacaacaccataatggaagacagtgtgccgg
cagaggtgtggaccagacacggagagaaaagagtgctcaaaccgaggtggatggacgccagagtttgttcagatcatgcggccctgaagtcattcaaggagtttgccgctgggaaaagaggagcggcttttggag
tgatggaagccctgggaacactgccaggacacatgacagagagattccaggaagccattgacaacctcgctgtgctcatgcgggcagagactggaagcaggccttacaaagccgcggcggcccaattgccggaga
ccctagagaccattatgcttttggggttgctgggaacagtctcgctgggaatctttttcgtcttgatgaggaacaagggcatagggaagatgggctttggaatggtgactcttggggccagcgcatggctcatgt
ggctctcggaaattgagccagccagaattgcatgtgtcctcattgttgtgttcctattgctggtggtgctcatacctgagccagaaaagcaaagatctccccaggacaaccaaatggcaatcatcatcatggtag
cagtaggtcttctgggcttgattaccgccaatgaactcggatggttggagagaacaaagagtgacctaagccatctaatgggaaggagagaggagggggcaaccataggattctcaatggacattgacctgcggc
cagcctcagcttgggccatctatgctgccttgacaactttcattacccagccgtccaacatgcagtgaccacttcatacaacaactactccttaatggcgatggccacgcaagctggagtgttgtttggtatgg
gcaaagggatgccattctacgcatgggactttggagtcccgctgctaatgataggttgctactcacaattaacacccctgaccctaatagtggccatcattttgctcgtggcgcactacatgtacttgatcccag
ggctgcaggcagcagctgcgcgtgctgcccagaagagaacggcagctggcatcatgaagaaccctgttgtggatggaatagtggtgactgacattgacacaatgacaattgacccccaagtggagaaaaagatgg
gacaggtgctactcatagcagtagccgtctccagcgccatactgtcgcggaccgcctggggggtgggggaggctggggccctgatcacagcggcaacttccactttgtgggaaggctctccgaacaagtactgga
actcctctacagccacttcactgtgtaacatttttaggggaagttacttggctggagcttctctaatctacacagtaacaagaaacgctggcttggtcaagagacgtgggggtggaacaggagagaccctgggag
agaaatggaaggcccgcttgaaccagatgtcggccctggagttctactcctacaaaaagtcaggcatcaccgaggtgtgcagagaagaggcccgccgcgccctcaaggacggtgtggcaacgggaggccatgctg
tgtcccgaggaagtgcaaagctgagatggttggtggagcggggatacctgcagccctatggaaaggtcattgatcttggatgtggcagaggggggctggagttactacgccgccaccatccgcaaagttcaagaag
tgaaaggatacacaaaaggaggccctggtcatgaagaacccatgttggtgcaaagctatgggtggaacatagtccgtcttaagagtggggtggacgtctttcatatggcggctgagccgtgtgacacgttgctgt
gtgacataggtgagtcatcatctagtcctgaagtggaagaagcacggacgctcagagtcctctccatggtgggggattggcttgaaaaaagaccaggagccttttgtataaaagtgttgtgcccatacaccagca
ctatgatggaaaccctggagcgactgcagcgtaggtatggggaggactggtcagagtgccactctcccgcaactctacacatgagatgtactgggtctctggagcgaaaagcaacaccataaaaagtgtgtcca
ccacgagccagctcctcttggggcgcatggacgggcccaggaggccagtgaaatatgaggaggatgtgaatctcggctctggcacgcgggctgtggtaagctgcgctgaagctcccaacatgaagatcattggta
accgcattgaaaggatccgcagtgagcacgcggaaacgtggttctttgacgagaacccacccatataggacatgggcttaccatggaagctatgaggccccacacaaggtcagcgtcctctctaataaacgggg
ttgtcaggctcctgtcaaaaccctgggatgtggtgactggagtcacaggaatagccatgaccgacaccacaccgtatggtcagcaaagagttttcaaggaaaaagtggacactagggtgccagacccccaagaag
gcactcgtcaggttatgagcatggtctcttcctggttgtggaaagagctaggcaaacacaaacggccacgagtctgtaccaaagaagagttcatcaacaaggttcgtagcaatgcagcattaggggcaatatttg
aagaggaaaaagagtggaagactgcagtggaagctgtgaacgatccaaggttctgggctctagtggacaaggaaagagagcaccacctgagaggagagtgccagagttgtgtgtacaacatgatgggaaaaagag
aaaagaaacaagggggaatttggaaaggccaagggcagccgcgccatctggtatatgtggctaggggctagatttctagagttcgaagcccttggattcttgaacgaggatcactggatgggggagagagaactcag
gaggtggtgttgaagggctgggattacaaagactcggatatgtcctagaagagatgagtcgcataccaggaggaaggatgtatgcagatgacactgctggctgggacacccgcatcagcaggtttgatctggaga
atgaagctctaatcaccaaccaaatggagaaagggcacagggccttggcattggccataatcaagtacacataccaaaacaaagtggtaaaggtccttagaccagctgaaaaagggaagacagttatggacatta
tttcgagacaagaccaaagggggagcggacaagttgtcacttacgctcttaacacatttaccaacctagtggtgcaactcattcggaatatggaggctgaggaagttctagagatgcaagacttgtggctgctgc
ggaggtcagagaaagtgaccaactggttgcagagcaacggatgggataggctcaaacgaatggcagtcagtggagatgattgcgttgtgaagccaattgatgataggtttgcacatgccctcaggttcttgaatg
atatgggaaaagttaggaaggacacacaagagtggaaaccctcaactggatgggacaactgggaagaagttccgttttgctcccaccacttcaacaagctccatctcaaggacgggaggtccattgtggttccct
gccgccaccaagatgaactgattggccgggcccgcgtctctccaggggcgggatggagcatccgggagactgcttgcctagcaaaatcatatgcgcaaatgtggcagctcctttatttccacagaagggacctcc
gactgatggccaatgccatttgttcatctgtgccagttgactgggttccaactgggagaactacctggtcaatccatggaaagggagaatggatgaccactgaagacatgcttgtggtgtggaacagagtgtgga
ttgaggagaacgaccacatggaagacaagacccagttacgaaatggacagacattccctatttgggaaaaaggaagacttgtggtgtggatctctcataggcacagaccgcgcaccacctgggctgagaaca
ttaaaaacacagtcaacatggtgcgcaggatcataggtgatgaagaaaagtacatggactacctatccacccaagttcgctacttgggtgaagaagggtctacacctggagtgctgtaagcaccaatcttagtgt
tgtcaggcctgctagtcagccacagcttggggaaagctgtgcagcctgtgacccccccaggagaagctgggaaaccaagcctatagtcaggccgagaacgccatggcacggaagaagccatgctgcctgtgagcc
cctcagaggacactgagtcaaaaaaccccacgcgcttggaggcgcaggatgggaaaagaaggtggcgaccttccccacccttcaatctggggcctgaactggagatcagctgtggatctccagaagagggactag
tggttagaggagacccccggaaaacgcaaaacagcatattgacgctgggaaagaccagagactccatgagtttccaccacgctggccgccaggcacagatcgccgaatagcggcggccggtgtggggaaatcca
tgggtct

Supplementary Figure 3. Complete genome sequences of WT, Permuted and CpG-recoded ZIKV variants (E+32CpG, E+102CpG and E/NS1+176CpG). CpG dinucleotides in regions encoding E and NS1 proteins (highlighted in yellow and green) are written in capitals and highlighted in bold.

>Permuted

agttgttgatctgtgtgaatcagactgcgacagttcgagtttgaagcgaaagctagcaacagtatcaacaggttttattttggatttggaaacgagagtttctggtcatgaaaaacccaaaaaagaaatccggag
gattccggattgtcaatatgctaaaacgcggagtagcccgtgtgagcccctttgggggcttgaagaggctgccagccggacttctgctgggtcatgggcccatcaggatggtcttggcgattctagcctttttga
gattcacggcaatcaagccatcactgggtctcatcaatagatggggttcagtggggaaaaaagaggctatggaaataataaagaagttcaagaaagatctggctgccatgctgagaataatcaatgctaggaagg
agaagaagagacgaggcgcagatactagtgtcggaattgttggcctcctgctgaccacagctatggcagcggaggtcactagacgtgggagtgcatactatatgtacttggacagaaacgacgctggggaggcca
tatcttttccaaccacattggggatgaataagtgttatatacagatcatggatcttggacacatgtgtgatgccaccatgagctatgaatgccctatgctggatgaggggtggaaccagatgacgtcgattgtt
ggtgcaacacgacgtcaacttgggttgtgtacggaacctgccatcacaaaaaaggtgaagcacggagatctagaagagctgtgacgctcccctcccattccactaggaagctgcaaacgcggtcgcaaacctggt
tggaatcaagagaatacacaaagcacttgattagagtcgaaaattggatattcaggaaccctggcttcgcgttagcagcagctgccatcgcttggcttttgggaagctcaacgagccaaaaagtcatatacttgg
tcatgatactgctgattgccccggcatacagcatcaggtgcataggagtcagcaatagagactttgtggaagggatgtctggtgggacatgggttgaCGtggtgttggaacatggaggatgtgtcacagtaatgg
cacaggacaagcccacagtCGacatagaactggttacaaccacagtcagcaatatggcagaagtgagatcctactgctatgaagcatcaatctCGgacatggcatcCGacagtCGctgccCGactcaaggtgagg
CGtatttagacaagcaatcagacacacaataCGtctgcaagagaacattagtggacaggggatggggaaatggatgtggtctctttggcaaagggagtcttgtgacttgCGctaagtttgcctgctctaagaaaa
tgacaggaaaaagtattcagccCGagaaccttgaatatCGaatcatgctctcagttcatggctcacagcacagtgggatgatCGtcaatgacactggacatgaaactgatgagaacagagCGaaagttgagataa
ccccaaactctcCGagggctgaggcaaccctgggaggctttgggagcttaggcctggactgtgaacctagaacaggacttgacttttCGacttatactacttgactatgaataacaaacattggttggttcaca
aggagtggtttcaCGacataccattgccatggcatgctggagcagacactggcacccccattggaacaacaaagaagcactggtggagtttaaggatgctcatgcaaagaggcaaaCGgtCGtggttctgggaa E
gccaggaaggagcagttcacacagcacttgcCGgagcactagaagctgagatggatggtgccaagggggaggctgtcttcaggccacttgaaatgcCGactgaaaatggataaactcagattaaagggCGtgtcat
actccttgtgcactgcagCGttcacattcaccaaaatcccagctgaaactctccatgggacagtgactgtggaggtccagtaCGcCGgtactgaCGgaccctgcaaggtaccCGcccagatggcagtggacatgc
aaactctgacacCGgttggcaggttaataacCGctaatccagtgattactgaaagcacCGagaactcaaagatgatgctggagcttgaccctccttttggggactCGtacattgtcattggCGtCGgagagaaga
agatcacccaccattggcacaggagtgggagcaccattgggaaagcatttgaggccaCGgtgagaggggccaagagaatggCGgtcttggggacacagcctgggatttggctcagttggtggagcacttaact
ctttgggcaaggggatccaccaaatttttggagcagctttcaagtcattgtttgggggaatgtcctggttctCGcaaattctcattggaaccctgctgatgtggttgggcctaaacacaaagaatggttccattt
cactcatgtgtttagctctaggaggagtgttgatctttttatccacagcagtgtctgctgatgtggggtgctCGgtggacttctcaaagaaggagaCGagatgCGgtacaggggtgttCGtctataaCGaCGttg
aagcctggagggacaggtacaagtaccatcctgactccccCGtagattggcagcagcagtcaagcaagcctgggaagatggtatctgtgggatctcctctgtttcaagaatggaaaacatcatgtggagatcag
tagaaggggagctcaaCGcaatcctggaagagaatggagttcaactgaCGgtCGttgtgggatctgtaaaaaaccccatgtggagaggtccacagagattgccCGtgcctgtgaaCGagctgccccaCGgctgga
aggcttgggggaaatCGtacttCGtcagagcagcaaagacaaataacagctttgtCGtggatggtgacacactgaaggaatgcccactcaaacatagagcatggaacagctttcttgtggaggatcatgggttCG NS1
gggtatttcacactagtgtctggctcaaggttagagaagattattcattagagtgtgatccagcCGttattggaacagctgttaagggaaaggaggctgtacacagtgatctaggctactggattgagagtgaga
agaatgacacatggaggctgaagagggcccatctgatCGagatgaaaacatgtgaatggccaaagtcccacacattgtggacagatggaatagaagagagtgatctgatcatacccaagtctttagctgggccac
tcagccatcacaataccagagagggctacaggacccaaatgaaagggccatggcacagtgaagagcttgaaattCGgtttgaggaatgcccaggcactaaggtccaCGtggaggaaacatgtggaacaagaggac
catctctgagatcaaccactgcaagCGgaagggtgatCGaggaatggtgctgcagggagtgcacaatgcccccactgtCGttcCGggctaaagatggctgttggtatggaatggagataaggcccaggaaagaac
cagaaagtaacttagtaaggtcaatggtgactgcaggatcaactgatcacatggatcacttctcccttggagtgcttgtgattctgctcatggtgcaggaagggctgaagaagagaatgaccacaaagatcatca
taagcacatcgatggcagtgctggtagctatgatcctgggaggattttcaatgagtgacctggctaagcttgcaattttgatgggtgccaccttcgcggaaatgaacactggaggagatgtagctcatctggcgc
tgatagcggcattcaaagtcagaccagcgttgctggtatctttcatcttcagagctaattggacaccccgtgaaagcatgctgctggccttggcctcgtgtcttttgcaaactgcgatctccgccttggaaggcg
acctgatggttctcatcaatggttttgctttggcctggttggcaatacgagcgatggttgttccacgcactgataacatcaccttggcaatcctggctgctctgacaccactggcccggggcacactgcttgtgg
cgtggagagcaggccttgctacttgcggggggtttatgctcctctctctgaagggaaaaggcagtgtgaagaagaacttaccatttgtcatggccctgggactaaccgctgtgaggctggtcgaccccatcaacg
tggtgggactgctgttgctcacaaggagtgggaagcggagctggcccccctagcgaagtactcacagctgttggcctgatatgcgcattggctggagggttcgccaaggcagatatagagatggctgggcccatgg
ccgcggtcggtctgctaattgtcagttacgtggtctcaggaaagagtgtggacatgtacattgaaagagcaggtgacatcacatgggaaaaagatgcggaagtcactggaaacagtccccggctcgatgtggcgc
tagatgagagtggtgatttctccctggtggaggatgacggtcccccatgagagagatcatactcaaggtggtcctgatgaccatctgtggcatgaacccaatagccataccctttgcagctggagcgtggtacg
tatacgtgaagactggaaaaaggagtggtgctctatgggatgtgcctgctcccaaggaagtaaaaaaggggagaccacagatggagtgtacagagtaatgactcgtagactgctaggttcaacacaagttggag
tgggagttatgcaagagggggtctttcacactatgtggcacgtcacaaaaggatccgcgctgagaagcggtgaagggagacttgatccatactggggagatgtcaagcaggatctggtgtcatactgtggtccat
ggaagctagatgccgcctgggacgggcacagcgaggtgcagctcttggccgtgccccccggagagagagcgaggaacatccagactctgcccggaatatttaagacaaaggatggggacattggagcggttgcgc
tggattacccagcaggaacttcaggatctccaatcctagacaagtgtgggagagtgataggactttatggcaatggggtcgtgatcaaaaatgggagttatgttagtgccatcacccaagggaggagggaggaag
agactcctgttgagtgcttcgagccttcgatgctgaagaagaagcagctaactgtcttagacttgcatcctggagctgggaaaaccaggagagttcttcctgaaatagtccgtgaagccataaaaacaagactcc
gtactgtgatcttagctccaaccagggttgtcgctgctgaaatggaggaagcccttagagggcttccagtgcgttatatgacaacagcagtcaatgtcacccactctggaacagaaatcgtcgacttaatgtgcc
atgccaccttcacttcacgtctactacagccaatcagagtccccaactataatctgtatattatggatgaggcccacttcacagatccctcaagtatagcagcaagaggatacatttcaacaagggttgagatgg
gcgaggcggctgccatcttcatgaccgccacgccaccaggaacccgtgacgcatttccggactccaactcaccaattatggacaccgaagtggaagtcccagagagagcctggagctcaggctttgattgggtga
cggatcattctggaaaaacagtttggtttgttccaagcgtgaggaacggcaatgagatcgcagcttgtctgacaaaggctggaaaacgggtcatacagctcagcagaaagacttttgagacagagttccagaaaa
caaaacatcaagagtgggactttgtcgtgacaactgacatttcagagatgggcgccaactttaaagctgaccgtgtcatagattccaggagatgcctaaagccggtcatacttgatggcgagagagtcattctgg
ctggacccatgcctgtcacacatgccagcgctgcccagaggagggggcgcataggcaggaatcccaacaaacctggagatgagtatctgtatggaggtgggtgcgcagagactgacgaagaccatgcacactggc
ttgaagcaagaatgctccttgacaatatttacctccaagatggcctcatagcctcgctctatcgacctgaggccgacaaagtagcagccattgagggagagttcaagcttaggacggagcaaaggaagacctttg
tggaactcatgaaaagaggagatcttcctgtttggctggcctatcaggttgcatctgccggaataacctacacagatagaagatggtgctttgatggcacgaccaacaacaccataatggaagacagtgtgccgg
cagaggtgtggaccagacacggagagaaaagagtgctcaaaccgaggtggatggacgccagagtttgttcagatcatgcggccctgaagtcattcaaggagtttgccgctgggaaaagaggagcggcttttggag
tgatggaagccctgggaacactgccaggacacatgacagagagattccaggaagccattgacaacctcgctgtgctcatgcgggcagagactggaagcaggccttacaaagccgcggcggcccaattgccggaga
ccctagagaccattatgcttttggggttgctgggaacagtctcgctgggaatctttttcgtcttgatgaggaacaagggcatagggaagatgggctttggaatggtgactcttggggccagcgcatggctcatgt
ggctctcggaaattgagccagccagaattgcatgtgtcctcattgttgtgttcctattgctggtggtgctcatacctgagccagaaaagcaaagatctccccaggacaaccaaatggcaatcatcatcatggtag
cagtaggtcttctgggcttgattaccgccaatgaactcggatggttggagagaacaaagagtgacctaagccatctaatgggaaggagagaggaggggggcaaccataggattctcaatggacattgacctgcggc
cagcctcagcttgggccatctatgctgccttgacaactttcattaccccagccgtccaacatgcagtgaccacttcatacaacaactactccttaatggcgatggccacgcaagctggagtgttgtttggtatgg
gcaaagggatgccattctacgcatgggactttggagtcccgctgctaatgataggttgctactcacaattaacacccctgaccctaatagtggccatcattttgctcgtggcgcactacatgtacttgatcccag
ggctgcaggcagcagctgcgcgtgctgcccagaagagaacggcagctggcatcatgaagaaccctgttgtggatggaatagtggtgactgacattgacacaatgacaattgacccccaagtggagaaaaagatgg
gacaggtgctactcatagcagtagccgtctccagcgccatactgtcgcggaccgcctggggtgggggaggctggggccctgatcacagcggcaacttccactttgtgggaaggctctccgaacaagtactgga
actcctctacagccacttcactgtgtaacatttttaggggaagttacttggctggagcttctctaatctacacagtaacaagaaacgctggcttggtcaagagacgtgggggtggaacaggagagaccctgggag
agaaatggaaggcccgcttgaaccagatgtcggccctggagttctactcctacaaaaagtcaggcatcaccgaggtgtgcagagaagaggcccgccgcgccctcaaggacggtgtggcaacggaggccatgctg
tgtcccgaggaagtgcaaagctgagatggttggtggagcggggatacctgcagccctatggaaaggtcattgatcttggatgtggcagaggggctggagttactacgccgccaccatccgcaaagttcaagaag
tgaaaggatacacaaaaggaggccctggtcatgaagaacccatgttggtgcaaagctatgggtggaacatagtccgtcttaagagtggggtggacgtctttcatatggcggctgagccgtgtgacacgttgctgt
gtgacataggtgagtcatcatctagtcctgaagtggaagaagcacggacgctcagagtcctctccatggtgggggattggcttgaaaaagaccaggagccttttgtataaaagtgttgtgcccatacaccagca
ctatgatggaaaccctggagcgactgcagcgtaggtatgggggaggactggtcagagtgccactctcccgcaactctacacatgagatgtactgggtctctggagcgaaaagcaacaccataaaaagtgtgtcca
ccacgagccagctcctcttggggcgcatggacgggcccaggaggccagtgaaatatgaggaggatgtgaatctcggctctggcacgcgggctgtggtaagctgcgctgaagctcccaacatgaagatcattggta
accgcattgaaaggatccgcagtgagcacgcggaaacgtggttctttgacgagaacccacccatataggacatgggcttaccatggaagctatgaggcccccacacaaggtcagcgtcctctctaataaacgggg
ttgtcaggctcctgtcaaaaccctgggatgtggtgactggagtcacaggaatagccatgaccgacaccacaccgtatggtcagcaaagagttttcaaggaaaaagtggacactagggtgccagacccccaagaag
gcactcgtcaggttatgagcatggtctcttcctggttgtggaaagagctaggcaaacacaaacggccacgagtctgtaccaaagaagagttcatcaacaaggttcgtagcaatgcagcattaggggcaatatttg
aagaggaaaaagagtggaagactgcagtggaagctgtgaacgatccaaggttctgggctctagtggacaaggaaagagagcaccacctgagaggagagtgccagagttgtgtgtacaacatgatgggaaaaagag
aaaagaaacaagggaatttggaaaggccaagggcagccgcgccatctggtatatgtggctaggggctagatttctagagttcgaagcccttggattcttgaacgaggatcactggatgggagagagaactcag
gaggtggtgttgaagggctgggattacaaagactcggatatgtcctagaagagatgagtcgcataccaggaggaaggatgtatgcagatgacactgctggctgggacacccgcatcagcaggtttgatctggaga
atgaagctctaatcaccaaccaaatggagaaagggcacagggccttggcattggccataatcaagtacacataccaaaacaaagtggtaaaggtccttagaccagctgaaaaaggaagacagttatggacatta
tttcgagacaagaccaaaggggggagcggacaagttgtcacttacgctcttaacacatttaccaacctagtggtgcaactcattcggaatatggaggctgaggaagttctagagatgcaagacttgtggctgctgc
ggaggtcagagaaagtgaccaactggttgcagagcaacggatgggataggctcaaacgaatggcagtcagtggagatgattgcgttgtgaagccaattgatgataggtttgcacatgccctcaggttcttgaatg
atatgggaaaagttaggaaggacacacaagagtggaaaccctcaactggatgggacaactgggaagaagttccgttttgctcccaccacttcaacaagctccatctcaaggacgggaggtccattgtggttccct
gccgccaccaagatgaactgattggccgggcccgcgtctctccaggggcgggatggagcatccgggagactgcttgcctagcaaaatcatatgcgcaaatgtggcagctcctttatttccacagaagggacctcc
gactgatggccaatgccatttgttcatctgtgccagttgactgggttccaactgggagaactacctggtcaatccatggaaagggagaatggatgaccactgaagacatgcttgtggtgtggaacagagtgtgga
ttgaggagaacgaccacatggaagacaagaccccagttacgaaatggacagacattccctatttgggaaaaagggaagacttgtggtgtggatctctcatagggcacagaccgcgcaccacctggctgagaaca
ttaaaaacacagtcaacatggtgcgcaggatcataggtgatgaagaaaagtacatggactacctatccacccaagttcgctacttgggtgaagaagggtctacacctggagtgctgtaagcaccaatcttagtgt
tgtcaggcctgctagtcagccacagcttggggaaagctgtgcagcctgtgacccccccaggagaagctgggaaaccaagcctatagtcaggccgagaacgccatggcacggaagaagccatgctgcctgtgagcc
cctcagaggacactgagtcaaaaaaccccacgcgcttggaggcgcaggatgggaaaagaaggtggcgaccttccccacccttcaatctggggcctgaactggagatcagctgtggatctccagaagagggactag
tggttagaggagacccccccggaaaacgcaaaacagcatattgacgctgggaaagaccagagactccatgagtttccaccacgctggccgccaggcacagatcgccgaatagcggcggcccggtgtggggaaatcca
tgggtct

Supplementary Figure 3 (continued).

>E+32CpG

agttgttgatctgtgtgaatcagactgcgacagttcgagtttgaagcgaaagctagcaacagtatcaacaggttttattttggatttggaaacgagagtttctggtcatgaaaaacccaaaaaagaaatccggag
gattccggattgtcaatatgctaaaacgcggagtagcccgtgtgagcccctttgggggcttgaagaggctgccagccggacttctgctgggtcatgggcccatcaggatggtcttggcgattctagcctttttga
gattcacggcaatcaagccatcactgggtctcatcaatagatggggttcagtggggaaaaaagaggctatggaaataataaagaagttcaagaaagatctggctgccatgctgagaataatcaatgctaggaagg
agaagaagagacgaggcgcagatactagtgtcggaattgttggcctcctgctgaccacagctatggcagcggaggtcactagacgtgggagtgcatactatatgtacttggacagaaacgacgctggggaggcca
tatcttttccaaccacattggggatgaataagtgttatatacagatcatggatcttggacacatgtgtgatgccaccatgagctatgaatgccctatgctggatgaggggggtggaaccagatgacgtcgattgtt
ggtgcaacacgacgtcaacttgggttgtgtacggaacctgccatcacaaaaaaggtgaagcacggagatctagaagagctgtgacgctcccctcccattccactaggaagctgcaaacgcggtcgcaaacctggt
tggaatcaagagaatacacaaagcacttgattagagtcgaaaattggatattcaggaaccctggcttcgcgttagcagcagctgccatcgcttggcttttgggaagctcaacgagccaaaaagtcatatacttgg
tcatgatactgctgattgccccggcatacagcatcaggtgcattggagtgagcaacagggactttgtggaagggatgtcaggtgggacttgggttgatgtagtcttggaacatggaggttgtgttacCGtcatgg
cacaagacaaacctacCGtagacatCGagctggtgaccacaacagtaagcaacatggctgaggtcagatcctactgctaCGaggcatctatttcagacatggcttctgacagcCGctgccCGacacaaggtgaag
cctaccttgacaagcaatcagacactcaatatgtctgcaaaagaactttggtggacagaggctggggaaatggatgtggacttttCGgcaaagggagccttgtgaCGtgCGCGaagtttgcatgttccaagaaaa
tgacCGgaaagagcatccagccagaaaatctggagtacCGaattatgctgtCGgttcatggctcccagcacagtgggatgatCGtgaaCGacacCGgacatgaaactgatgagaatCGagCGaaggtagagatta
CGcCGaattcaccaagagcagaagCGactctggggggttttggaagcctCGgacttgattgtgaaccaagaacaggccttgactttcCGatttgtattacttaactatgaacaacaaacactggttagtgcaca
aggagtggtttcatgatattccattaccttggcaCGctggggcCGacactggaactccacactggaataacaaagaagcactggtggagttcaaggaCGcacatgccaaaCGgcaaactgtCGtggttcttggga **E**
gtcaagaaggagcagtccacacCGcccttgCGggCGcacttgaggctgagatggatggCGCGaaaggaaggctgtCGtctggccacttgaaatgtCGcctgaaaatggacaaactgagattgaagggagtctcat
actcattgtgcacCGcagCGttcacattcacaaagatccCGgctgaaacactacaCGggacCGtaacagtggaggttcaataCGcagggacagatggaccttgcaaggtaccagctcagatggCGgtCGacatgc
aaactctgaCGccagttggcaggttgatcacagcaaatccCGtaatcaCGgaaagcacCGagaactCGaagatgatgctCGaacttgatccaccatttggCGactcttacattgttataggagtaggggagaaga
agattaCGcatcattggcacaggagtggaagcacaattggaaaagCGtttgaagctactgtaagaggCGCGaagCGaatggCGgtattgggagacaCGgcctgggactttggatcCGtCGgaggCGctcttaatt
cattgggcaaaggcatccatcaaattttggCGCGgctttcaaatcattgttCGgaggaatgtcctggttctCGcaaatacttatCGgaaCGttgcttatgtggttgggtctcaacacaaagaatggatcaattt
ccctgatgtgcttggccttgggggagtttgatcttcttatctacagctgtctCGgctgatgtggggtgctCGgtggacttctcaaagaaggagaCGagatgCGgtacaggggtgttCGtctataaCGaCGttg
aagcctggagggacaggtacaagtaccatcctgactcccccCGtagattggcagcagcagtcaagcaagcctgggaagatggtatctgtgggatctcctctgtttcaagaatggaaaacatcatgtggagatcag
tagaaggggagctcaaCGcaatcctggaagagaatggagttcaactgaCGgtCGttgtgggatctgtaaaaaaccccatgtggagaggtccacagagattgccCGtgcctgtgaaCGagctgccccaCGgctgga
aggcttggggaaatCGtacttCGtcagagcagcaaagacaaataacagctttgtCGtggatggtgacacactgaaggaatgcccactcaaacatagagcatggaacagctttcttgtggaggatcatgggttCG **NS1**
gggtatttcacactagtgtctggctcaaggttagagaagattattcattagagtgtgatccagcCGttattggaacagctgttaagggaaaggaggctgtacacagtgatctaggctactggattgagagtgaga
agaatgacacatggaggctgaagagggcccatctgatCGagatgaaaacatgtgaatggccaaagtcccacacattgtggacagatggaatagaagagagtgatctgatcatacccaagtctttagctgggccac
tcagccatcacaataccagagagggctacaggacccaaatgaaagggccatggcacagtgaagagcttgaaattCGgtttgaggaatgcccaggcactaaggtccaCGtggaggaaacatgtggaacaagaggac
catctctgagatcaaccactgcaagCGgaagggtgatCGaggaatggtgctgcagggagtgcacaatgcccccactgtCGttcCGggctaaagatggctgttggtatggaatggagataaggcccaggaaagaac
cagaaagtaacttagtaaggtcaatggtgactgcaggatcaactgatcacatggatcacttctcccttggagtgcttgtgattctgctcatggtgcaggaagggctgaagaagagaatgaccacaaagatcatca
taagcacatcgatggcagtgctggtagctatgatcctgggaggattttcaatgagtgacctggctaagcttgcaattttgatgggtgccaccttcgcggaaatgaacactggaggagatgtagctcatctggcgc
tgatagcggcattcaaagtcagaccagcgttgctggtatctttcatcttcagagctaattggacaccccgtgaaagcatgctgctggccttggcctcgtgtcttttgcaaactgcgatctccgccttggaaggcg
acctgatggttctcatcaatggttttgctttggcctggttggcaatacgagcgatggttgttccacgcactgataacatcaccttggcaatcctggctgctctgacaccactggcccggggcacactgcttgtgg
cgtggagagcaggccttgctacttgcggggggtttatgctcctctctctgaagggaaaaggcagtgtgaagaagaacttaccatttgtcatggccctgggactaaccgctgtgaggctggtcgaccccatcaacg
tggtgggactgctgttgctcacaaggagtgggaagcggagctggcccccctagcgaagtactcacagctgttggcctgatatgcgcattggctggagggttcgccaaggcagatatagagatggctgggcccatgg
ccgcggtcggtctgctaattgtcagttacgtggtctcaggaaagagtgtggacatgtacattgaaagagcaggtgacatcacatgggaaaaagatgcggaagtcactggaaacagtccccggctcgatgtggcgc
tagatgagagtggtgatttctccctggtggaggatgacggtcccccatgagagagatcatactcaaggtggtcctgatgaccatctgtggcatgaacccaatagccataccctttgcagctggagcgtggtacg
tatacgtgaagactggaaaaaggagtggtgctctatgggatgtgcctgctcccaaggaagtaaaaaaggggggagaccacagatggagtgtacagagtaatgactcgtagactgctaggttcaacacaagttggag
tgggagttatgcaagagggggtctttcacactatgtggcacgtcacaaaaggatccgcgctgagaagcggtgaagggagacttgatccatactggggagatgtcaagcaggatctggtgtcatactgtggtccat
ggaagctagatgccgcctgggacgggcacagcgaggtgcagctcttggccgtgccccccggagagagagcgaggaacatccagactctgcccggaatatttaagacaaaggatggggacattggagcggttgcgc
tggattacccagcaggaacttcaggatctccaatcctagacaagtgtgggagagtgataggactttatggcaatggggtcgtgatcaaaaatgggagttatgttagtgccatcacccaagggaggagggaggaag
agactcctgttgagtgcttcgagccttcgatgctgaagaagaagcagctaactgtcttagacttgcatcctggagctgggaaaaccaggagagttcttcctgaaatagtccgtgaagccataaaaacaagactcc
gtactgtgatcttagctccaaccagggttgtcgctgctgaaatggaggaagcccttagagggcttccagtgcgttatatgacaacagcagtcaatgtcacccactctggaacagaaatcgtcgacttaatgtgcc
atgccaccttcacttcacgtctactacagccaatcagagtccccaactataatctgtatattatggatgaggcccacttcacagatccctcaagtatagcagcaagaggatacatttcaacaagggttgagatgg
gcgaggcggctgccatcttcatgaccgccacgccaccaggaacccgtgacgcatttccggactccaactcaccaattatggacaccgaagtggaagtcccagagagagcctggagctcaggctttgattgggtga
cggatcattctggaaaaacagtttggtttgttccaagcgtgaggaacggcaatgagatcgcagcttgtctgacaaaggctggaaaacgggtcatacagctcagcagaaagacttttgagacagagttccagaaaa
caaaacatcaagagtgggactttgtcgtgacaactgacatttcagagatgggcgccaactttaaagctgaccgtgtcatagattccaggagatgcctaaagccggtcatacttgatggcgagagagtcattctgg
ctggacccatgcctgtcacacatgccagcgctgcccagaggagggggcgcataggcaggaatcccaacaaacctggagatgagtatctgtatggaggtgggtgcgcagagactgacgaagaccatgcacactggc
ttgaagcaagaatgctccttgacaatatttacctccaagatggcctcatagcctcgctctatcgacctgaggccgacaaagtagcagccattgagggagagttcaagcttaggacggagcaaaggaagacctttg
tggaactcatgaaaagaggagatcttcctgtttggctggcctatcaggttgcatctgccggaataacctacacagatagaagatggtgctttgatggcacgaccaacaacaccataatggaagacagtgtgccgg
cagaggtgtggaccagacacggagagaaaagagtgctcaaaccgaggtggatggacgccagagtttgttcagatcatgcggccctgaagtcattcaaggagtttgccgctgggaaaagaggagcggcttttggag
tgatggaagccctgggaacactgccaggacacatgacagagagattccaggaagccattgacaacctcgctgtgctcatgcgggcagagactggaagcaggccttacaaagccgcggcggcccaattgccggaga
ccctagagaccattatgcttttggggttgctgggaacagtctcgctgggaatctttttcgtcttgatgaggaacaagggcatagggaagatgggctttggaatggtgactcttggggccagcgcatggctcatgt
ggctctcggaaattgagccagccagaattgcatgtgtcctcattgttgtgttcctattgctggtggtgctcatacctgagccagaaaagcaaagatctccccaggacaaccaaatggcaatcatcatcatggtag
cagtaggtcttctgggcttgattaccgccaatgaactcggatggttggagagaacaaagagtgacctaagccatctaatgggaaggagagaggaggggggcaaccataggattctcaatggacattgacctgcggc
cagcctcagcttgggccatctatgctgccttgacaactttcattaccccagccgtccaacatgcagtgaccacttcatacaacaactactccttaatggcgatggccacgcaagctggagtgttgtttggtatgg
gcaaagggatgccattctacgcatgggactttggagtcccgctgctaatgataggttgctactcacaattaacacccctgaccctaatagtggccatcattttgctcgtggcgcactacatgtacttgatcccag
ggctgcaggcagcagctgcgcgtgctgcccagaagagaacggcagctggcatcatgaagaaccctgttgtggatggaatagtggtgactgacattgacacaatgacaattgacccccaagtggagaaaaagatgg
gacaggtgctactcatagcagtagccgtctccagcgccatactgtcgcggaccgcctgggggtgggggaggctggggccctgatcacagcggcaacttccactttgtgggaaggctctccgaacaagtactgga
actcctctacagccacttcactgtgtaacattttaggggaagttacttggctggagcttctctaatctacacagtaacaagaaacgctggcttggtcaagagacgtgggggtggaacaggagagaccctgggag
agaaatggaaggcccgcttgaaccagatgtcggccctggagttctactcctacaaaaagtcaggcatcaccgaggtgtgcagagaagaggcccgccgcgccctcaaggacggtgtggcaacgggaggccatgctg
tgtcccgaggaagtgcaaagctgagatggttggtggagcggggatacctgcagccctatggaaaggtcattgatcttggatgtggcagaggggggctggagttactacgccgccaccatccgcaaagttcaagaag
tgaaaggatacacaaaaggaggccctggtcatgaagaacccatgttggtgcaaagctatgggtggaacatagtccgtcttaagagtggggtggacgtctttcatatggcggctgagccgtgtgacacgttgctgt
gtgacataggtgagtcatcatctagtcctgaagtggaagaagcacggacgctcagagtcctctccatggtgggggattggcttgaaaaaagaccaggagcctttgtataaaagtgttgtgcccatacaccagca
ctatgatggaaaccctggagcgactgcagcgtaggtatgggggaggactggtcagagtgccactctcccgcaactctacacatgagatgtactgggtctctggagcgaaaagcaacaccataaaaagtgtgtcca
ccacgagccagctcctcttggggcgcatggacggcccaggaggccagtgaaatatgaggaggatgtgaatctcggctctggcacgcgggctgtggtaagctgcgctgaagctcccaacatgaagatcattggta
accgcattgaaaggatccgcagtgagcacgcggaaacgtggttctttgacgagaacccacccatataggacatgggcttaccatggaagctatgaggcccccacacaagggtcagcgtcctctctaataaacgggg
ttgtcaggctcctgtcaaaaccctgggatgtggtgactggagtcacaggaatagccatgaccgacaccacaccgtatggtcagcaaagagttttcaaggaaaaagtggacactagggtgccagacccccaagaag
gcactcgtcaggttatgagcatggtctcttcctggttgtggaaagagctaggcaaacacaaacggccacgagtctgtaccaaagaagagttcatcaacaaggttcgtagcaatgcagcattaggggcaatatttg
aagaggaaaaagagtggaagactgcagtggaagctgtgaacgatccaaggttctgggctctagtggacaaggaaagagagcaccacctgagaggagagtgccagagttgtgtgtacaacatgatgggaaaaagag
aaaagaaacaagggaatttggaaaggccaagggcagccgcgccatctggtatatgtggctaggggctagatttctagagttcgaagcccttggattcttgaacgaggatcactggatgggagagagaactcag
gaggtggtgttgaagggctgggattacaaagactcggatatgtcctagaagagatgagtcgcataccaggaggaaggatgtatgcagatgacactgctggctgggacacccgcatcagcaggtttgatctggaga
atgaagctctaatcaccaaccaaatggagaaagggcacagggccttggcattggccataatcaagtacacataccaaaacaaagtggtaaaggtccttagaccagctgaaaaaggaagacagttatggacatta
tttcgagacaagaccaaaggggggagcggacaagttgtcacttacgctcttaacacatttaccaacctagtggtgcaactcattcggaatatggaggctgaggaagttctagagatgcaagacttgtggctgctgc
ggaggtcagagaaagtgaccaactggttgcagagcaacggatgggataggctcaaacgaatggcagtcagtggagatgattgcgttgtgaagccaattgatgataggtttgcacatgccctcaggttcttgaatg
atatgggaaaagttaggaaggacacacaagagtggaaaccctcaactggatgggacaactgggaagaagttccgttttgctcccaccacttcaacaagctccatctcaaggacgggaggtccattgtggttccct
gccgccaccaagatgaactgattggccgggcccgcgtctctccaggggcgggatggagcatccgggagactgcttgcctagcaaaatcatatgcgcaaatgtggcagctcctttatttccacagaagggacctcc
gactgatggccaatgccatttgttcatctgtgccagttgactgggttccaactgggagaactacctggtcaatccatggaaagggagaatggatgaccactgaagacatgcttgtggtgtggaacagagtgtgga
ttgaggagaacgaccacatggaagacaagaccccagttacgaaatggacagacattccctatttgggaaaaagggaagacttgtggtgtggatctctcatagggcacagaccgcgcaccacctgggctgagaaca
ttaaaaacacagtcaacatggtgcgcaggatcataggtgatgaagaaaagtacatggactacctatccacccaagttcgctacttgggtgaagaagggtctacacctggagtgctgtaagcaccaatcttagtgt
tgtcaggcctgctagtcagccacagcttggggaaagctgtgcagcctgtgaccccccaggagaagctgggaaaccaagcctatagtcaggccgagaacgccatggcacggaagaagccatgctgcctgtgagcc
cctcagaggacactgagtcaaaaaaccccacgcgcttggaggcgcaggatgggaaaagaaggtggcgaccttccccacccttcaatctggggcctgaactggagatcagctgtggatctccagaagagggactag
tggttagaggagacccccccggaaaacgcaaaacagcatattgacgctgggaaagaccagagactccatgagtttccaccacgctggccgccaggcacagatcgccgaatagcggcggccggtgtggggaaatcca
tgggtct

Supplementary Figure 3 (continued).

>E+102CpG

agttgttgatctgtgtgaatcagactgcgacagttcgagtttgaagcgaaagctagcaacagtatcaacaggttttattttggatttggaaacgagagtttctggtcatgaaaaacccaaaaaagaaatccggag
gattccggattgtcaatatgctaaaacgcggagtagcccgtgtgagcccctttgggggcttgaagaggctgccagccggacttctgctgggtcatgggcccatcaggatggtcttggcgattctagcctttttga
gattcacggcaatcaagccatcactgggtctcatcaatagatggggttcagtggggaaaaaagaggctatggaaataataaagaagttcaagaaagatctggctgccatgctgagaataatcaatgctaggaagg
agaagaagagacgaggcgcagatactagtgtcggaattgttggcctcctgctgaccacagctatggcagcggaggtcactagacgtgggagtgcatactatatgtacttggacagaaacgacgctggggaggcca
tatcttttccaaccacattggggatgaataagtgttatatacagatcatggatcttggacacatgtgtgatgccaccatgagctatgaatgccctatgctggatgaggggggtggaaccagatgacgtcgattgtt
ggtgcaacacgacgtcaacttgggttgtgtacggaacctgccatcacaaaaaaggtgaagcacggagatctagaagagctgtgacgctcccctcccattccactaggaagctgcaaacgcggtcgcaaacctggt
tggaatcaagagaatacacaaagcacttgattagagtcgaaaattggatattcaggaaccctggcttcgcgttagcagcagctgccatcgcttggcttttgggaagctcaacgagccaaaaagtcatatacttgg
tcatgatactgctgattgccccggcatacagcatcCGgtgcatCGgCGtaagcaacagagatttCGtCGaaggcatgtCGggCGgcaCGtgggttgaCGtCGtcttggaacaCGgaggttgtgtaaCGgtcatgg
cacaagacaaacccaCGgttgacatagaacttgtgaCGacaacCGtcagcaacatggcCGaagttCGatcttactgctatgaagcctccatttcCGacatggcatctgatagtCGatgtcCGaCGcaaggCGaag
catatcttgacaaacaatCGgacactcaataCGtatgcaaaCGaaCGttagtggatCGaggatggggaaaCGgatgCGgactattCGgaaaagggagtctCGtaaCGtgtgctaaattCGCGtgttCGaaaaaaa
tgaCGgggaagagcatacaaccagagaatctagaatacCGtatcatgctttCGgttcaCGgatctcaacacagCGgaatgatCGtgaatgacaCGggacatgaaaCGgaCGaaaatCGCGCGaaagttgagataa
ctcctaattcacctCGagCGgaagcaaCGctCGgtggtttCGgaagtcttggactagattgtgaacCGCGaacCGgtctCGatttttCGgatttgtattatttgaCGatgaacaacaagcattggttggttcaca
aagaatggtttcaCGacattccattgccttggcatgCGggCGCGgataCGggtaCGcCGcattggaacaacaaagaagcactagttgagttcaaagaCGcacatgCGaaaCGtcaaacagtCGtagtcctaggga
gtcaagaaggCGCGgttcacacCGCGctCGcCGgCGCGctagaagctgagatggaCGgCGCGaagggaCGactgtCGtctggtcatttgaaatgtCGactgaaaatggacaaacttCGattaaaaggtgtatcat **E**
attCGttgtgcaCGgCGgctttcactttcacaaagatccCGgcagagacacttcaCGgcacCGtgacCGtCGaggtccaatatgCGggcacCGaCGgacCGtgtaaagttccCGCGcagatggcCGtggacatgc
aaaCGctgaCGccagtCGgtaggttgatcaCGgCGaatcCGgtgatcaCGgaaagcaCGgaaaactCGaagatgatgctagaacttgatcCGccattCGgagactcttatattgtaattggCGtaggCGagaaaa
aaataaCGcatcattggcatCGaagCGggagcaCGattggaaaagcatttgaagCGactgttCGaggCGcaaaaCGaatggcagttttgggCGacacCGCGtgggacttCGgatcagtCGgCGgagcactgaatt
CGttgggaaaaggaattcatcaaattttCGgCGcagCGttcaaatcattgtttggaggaatgtCGtggttttcacaaattctcataggaaCGttgctgatgtggttgggtctgaacacaaagaaCGgatcaattt
CGctcatgtgtttagCGttgggCGgtgtgttgatattttgtcaactgCGgtttctgctgatgtggggtgctCGgtggacttctcaaagaaggagaCGagatgCGgtacaggggtgttCGtctataaCGaCGttg
aagcctggagggacaggtacaagtaccatcctgactccccCGtagattggcagcagcagtcaagcaagcctgggaagatggtatctgtgggatctcctctgtttcaagaatggaaaacatcatgtggagatcag
tagaaggggagctcaaCGcaatcctggaagagaatggagttcaactgaCGgtCGttgtgggatctgtaaaaaaccccatgtggagaggtccacagagattgccCGtgcctgtgaaCGagctgccccaCGgctgga
aggcttggggaaatCGtacttCGtcagagcagcaaagacaaataacagctttgtCGtggatggtgacacactgaaggaatgcccactcaaacatagagcatggaacagctttcttgtggaggatcatgggttCG
gggtatttcacactagtgtctggctcaaggttagagaagattattcattagagtgtgatccagcCGttattggaacagctgttaagggaaaggaggctgtacacagtgatctaggctactggattgagagtgaga **NS1**
agaatgacacatggaggctgaagagggcccatctgatCGagatgaaaacatgtgaatggccaaagtcccacacattgtggacagatggaatagaagagagtgatctgatcatacccaagtctttagctgggccac
tcagccatcacaataccagagagggctacaggacccaaatgaaagggccatggcacagtgaagagcttgaaattCGgtttgaggaatgcccaggcactaaggtccaCGtggaggaaacatgtggaacaagaggac
catctctgagatcaaccactgcaagCGgaagggtgatCGaggaatggtgctgcagggagtgcacaatgcccccactgtCGttcCGggctaaagatggctgttggtatggaatggagataaggcccaggaaagaac
cagaaagtaacttagtaaggtcaatggtgactgcaggatcaactgatcacatggatcacttctcccttggagtgcttgtgattctgctcatggtgcaggaagggctgaagaagagaatgaccacaaagatcatca
taagcacatcgatggcagtgctggtagctatgatcctgggaggattttcaatgagtgacctggctaagcttgcaattttgatgggtgccaccttcgcggaaatgaacactggaggagatgtagctcatctggcgc
tgatagcggcattcaaagtcagaccagcgttgctggtatctttcatcttcagagctaattggacaccccgtgaaagcatgctgctggccttggcctcgtgtcttttgcaaactgcgatctccgccttggaaggcg
acctgatggttctcatcaatggttttgctttggcctggttggcaatacgagcgatggttgttccacgcactgataacatcaccttggcaatcctggctgctctgacaccactggcccggggcacactgcttgtgg
cgtggagagcaggccttgctacttgcggggggtttatgctcctctctctgaagggaaaaggcagtgtgaagaagaacttaccatttgtcatggccctgggactaaccgctgtgaggctggtcgaccccatcaacg
tggtgggactgctgttgctcacaaggagtgggaagcggagctggcccccctagcgaagtactcacagctgttggcctgatatgcgcattggctggagggttcgccaaggcagatatagagatggctgggcccatgg
ccgcggtcggtctgctaattgtcagttacgtggtctcaggaaagagtgtggacatgtacattgaaagagcaggtgacatcacatgggaaaaagatgcggaagtcactggaaacagtccccggctcgatgtggcgc
tagatgagagtggtgatttctccctggtggaggatgacggtccccccatgagagagatcatactcaaggtggtcctgatgaccatctgtggcatgaacccaatagccatacccttttgcagctggagcgtggtacg
tatacgtgaagactggaaaaaggagtggtgctctatgggatgtgcctgctcccaaggaagtaaaaaaggggggagaccacagatggagtgtacagagtaatgactcgtagactgctaggttcaacacaagttggag
tgggagttatgcaagagggggtctttcacactatgtggcacgtcacaaaaggatccgcgctgagaagcggtgaagggagacttgatccatactggggagatgtcaagcaggatctggtgtcatactgtggtccat
ggaagctagatgccgcctgggacgggcacagcgaggtgcagctcttggccgtgccccccggagagagagcgaggaacatccagactctgcccggaatatttaagacaaaggatggggacattggagcggttgcgc
tggattacccagcaggaacttcaggatctccaatcctagacaagtgtgggagagtgataggactttatggcaatggggtcgtgatcaaaaatgggagttatgttagtgccatcacccaagggaggagggaggaag
agactcctgttgagtgcttcgagccttcgatgctgaagaagaagcagctaactgtcttagacttgcatcctggagctgggaaaaccaggagagttcttcctgaaatagtccgtgaagccataaaaacaagactcc
gtactgtgatcttagctccaaccagggttgtcgctgctgaaatggaggaagcccttagagggcttccagtgcgttatatgacaacagcagtcaatgtcacccactctggaacagaaatcgtcgacttaatgtgcc
atgccaccttcacttcacgtctactacagccaatcagagtccccaactataatctgtatattatggatgaggcccacttcacagatccctcaagtatagcagcaagaggatacatttcaacaagggttgagatgg
gcgaggcggctgccatcttcatgaccgccacgccaccaggaacccgtgacgcatttccggactccaactcaccaattatggacaccgaagtggaagtcccagagagagcctggagctcaggctttgattgggtga
cggatcattctggaaaaacagtttggtttgttccaagcgtgaggaacggcaatgagatcgcagcttgtctgacaaaggctggaaaacgggtcatacagctcagcagaaagacttttgagacagagttccagaaaa
caaaacatcaagagtgggactttgtcgtgacaactgacatttcagagatgggcgccaactttaaagctgaccgtgtcatagattccaggagatgcctaaagccggtcatacttgatggcgagagagtcattctgg
ctggacccatgcctgtcacacatgccagcgctgcccagaggagggggcgcataggcaggaatcccaacaaacctggagatgagtatctgtatggaggtgggtgcgcagagactgacgaagaccatgcacactggc
ttgaagcaagaatgctccttgacaatatttacctccaagatggcctcatagcctcgctctatcgacctgaggccgacaaagtagcagccattgagggagagttcaagcttaggacggagcaaaggaagacctttg
tggaactcatgaaaagaggagatcttcctgtttggctggcctatcaggttgcatctgccggaataacctacacagatagaagatggtgctttgatggcacgaccaacaacaccataatggaagacagtgtgccgg
cagaggtgtggaccagacacggagagaaaagagtgctcaaaccgaggtggatggacgccagagtttgttcagatcatgcggccctgaagtcattcaaggagtttgccgctgggaaaagaggagcggcttttggag
tgatggaagccctgggaacactgccaggacacatgacagagagattccaggaagccattgacaacctcgctgtgctcatgcgggcagagactggaagcaggccttacaaagccgcggcggcccaattgccggaga
ccctagagaccattatgcttttggggttgctgggaacagtctcgctgggaatctttttcgtcttgatgaggaacaagggcatagggaagatgggctttggaatggtgactcttggggccagcgcatggctcatgt
ggctctcggaaattgagccagccagaattgcatgtgtcctcattgttgtgttcctattgctggtggtgctcatacctgagccagaaaagcaaagatctccccaggacaaccaaatggcaatcatcatcatggtag
cagtaggtcttctgggcttgattaccgccaatgaactcggatggttggagagaacaaagagtgacctaagccatctaatgggaaggagagaggagggggcaaccataggattctcaatggacattgacctgcggc
cagcctcagcttgggccatctatgctgccttgacaactttcattaccccagccgtccaacatgcagtgaccacttcatacaacaactactccttaatggcgatggccacgcaagctggagtgttgtttggtatgg
gcaaagggatgccattctacgcatgggactttggagtcccgctgctaatgataggttgctactcacaattaacacccctgaccctaatagtggccatcattttgctcgtggcgcactacatgtacttgatcccag
ggctgcaggcagcagctgcgcgtgctgcccagaagagaacggcagctggcatcatgaagaaccctgttgtggatggaatagtggtgactgacattgacacaatgacaattgacccccaagtggagaaaaagatgg
gacaggtgctactcatagcagtagccgtctccagcgccatactgtcgcggaccgcctggggggtgggggaggctggggccctgatcacagcggcaacttccactttgtgggaaggctctccgaacaagtactgga
actcctctacagccacttcactgtgtaacattttttaggggaagttacttggctggagcttctctaatctacacagtaacaagaaacgctggcttggtcaagagacgtgggggtggaacaggagagaccctgggag
agaaatggaaggcccgcttgaaccagatgtcggccctggagttctactcctacaaaaagtcaggcatcaccgaggtgtgcagagaagaggcccgccgcgccctcaaggacggtgtggcaacgggaggccatgctg
tgtcccgaggaagtgcaaagctgagatggttggtggagcggggatacctgcagccctatggaaaggtcattgatcttggatgtggcagaggggggctggagttactacgccgccaccatccgcaaagttcaagaag
tgaaaggatacacaaaaggaggccctggtcatgaagaacccatgttggtgcaaagctatgggtggaacatagtccgtcttaagagtggggtggacgtctttcatatggcggctgagccgtgtgacacgttgctgt
gtgacataggtgagtcatcatctagtcctgaagtggaagaagcacggacgctcagagtcctctccatggtgggggattggcttgaaaaaagaccaggagccttttgtataaaagtgttgtgcccatacaccagca
ctatgatggaaaccctggagcgactgcagcgtaggtatggggaggactggtcagagtgccactctcccgcaactctacacatgagatgtactgggtctctggagcgaaaagcaacaccataaaaagtgtgtcca
ccacgagccagctcctcttggggcgcatggacgggcccaggaggccagtgaaatatgaggaggatgtgaatctcggctctggcacgcgggctgtggtaagctgcgctgaagctcccaacatgaagatcattggta
accgcattgaaaggatccgcagtgagcacgcggaaacgtggttctttgacgagaaccacccatataggacatgggcttaccatggaagctatgaggcccccacacaagggtcagcgtcctctctaataaacgggg
ttgtcaggctcctgtcaaaaccctgggatgtggtgactggagtcacaggaatagccatgaccgacaccacaccgtatggtcagcaaagagttttcaaggaaaaagtggacactagggtgccagacccccaagaag
gcactcgtcaggttatgagcatggtctcttcctggttgtggaaagagctaggcaaacacaaacggccacgagtctgtaccaaagaagagttcatcaacaaggttcgtagcaatgcagcattaggggcaatatttg
aagaggaaaaagagtggaagactgcagtggaagctgtgaacgatccaaggttctgggctctagtggacaaggaaagagagcaccacctgagaggagagtgccagagttgtgtgtacaacatgatgggaaaaagag
aaaagaaacaagggggaatttggaaaggccaagggcagccgcgccatctggtatatgtggctaggggctagatttctagagttcgaagcccttggattcttgaacgaggatcactggatggggagagagaactcag
gaggtggtgttgaagggctgggattacaaagactcggatatgtcctagaagagatgagtcgcataccaggaggaaggatgtatgcagatgacactgctggctgggacacccgcatcagcaggtttgatctggaga
atgaagctctaatcaccaaccaaatggagaaagggcacagggccttggcattggccataatcaagtacacataccaaaacaaagtggtaaaggtccttagaccagctgaaaaagggaagacagttatggacatta
tttcgagacaagaccaaagggggagcggacaagttgtcacttacgctcttaacacatttaccaacctagtggtgcaactcattcggaatatggaggctgaggaagttctagagatgcaagacttgtggctgctgc
ggaggtcagagaaagtgaccaactggttgcagagcaacggatgggataggctcaaacgaatggcagtcagtggagatgattgcgttgtgaagccaattgatgataggtttgcacatgccctcaggttcttgaatg
atatgggaaaagttaggaaggacacacaagagtggaaaccctcaactggatgggacaactgggaagaagttccgttttgctcccaccacttcaacaagctccatctcaaggacgggaggtccattgtggttccct
gccgccaccaagatgaactgattggccgggcccgcgtctctccaggggcgggatggagcatccgggagactgcttgcctagcaaaatcatatgcgcaaatgtggcagctcctttatttccacagaagggacctcc
gactgatggccaatgccatttgttcatctgtgccagttgactgggttccaactgggagaactacctggtcaatccatggaaagggagaatggatgaccactgaagacatgcttgtggtgtggaacagagtgtgga
ttgaggagaacgaccacatggaagacaagaccccagttacgaaatggacagacattccctatttgggaaaaagggaagacttgtggtgtggatctctcatagggcacagaccgcgcaccacctgggctgagaaca
ttaaaaacacagtcaacatggtgcgcaggatcataggtgatgaagaaaagtacatggactacctatccacccaagttcgctacttgggtgaagaagggtctacacctggagtgctgtaagcaccaatcttagtgt
tgtcaggcctgctagtcagccacagcttggggaaagctgtgcagcctgtgacccccccaggagaagctgggaaaccaagcctatagtcaggccgagaacgccatggcacggaagaagccatgctgcctgtgagcc
cctcagaggacactgagtcaaaaaaccccacgcgcttggaggcgcaggatggaaaagaaggtggcgaccttccccacccttcaatctggggcctgaactggagatcagctgtggatctccagaagagggactag
tggttagaggagacccccccggaaaacgcaaaacagcatattgacgctgggaaagaccagagactccatgagtttccaccacgctggccgccaggcacagatcgccgaatagcggcggccggtgtggggaaatcca
tgggtct

Supplementary Figure 3 (continued).

>E/NS1+176CpG

```
agttgttgatctgtgtgaatcagactgcgacagttcgagtttgaagcgaaagctagcaacagtatcaacaggttttattttggatttggaaacgagagtttctggtcatgaaaaacccaaaaaagaaatccggag
gattccggattgtcaatatgctaaaacgcggagtagcccgtgtgagcccctttgggggcttgaagaggctgccagccggacttctgctgggtcatgggcccatcaggatggtcttggcgattctagccttttga
gattcacggcaatcaagccatcactgggtctcatcaatagatggggttcagtggggaaaaaagaggctatggaaataataaagaagttcaagaaagatctggctgccatgctgagaataatcaatgctaggaagg
agaagaagagacgaggcgcagatactagtgtcggaattgttggcctcctgctgaccacagctatggcagcggaggtcactagacgtgggagtgcatactatatgtacttggacagaaacgacgctggggaggcca
tatcttttccaaccacattggggatgaataagtgttatatacagatcatggatcttggacacatgtgtgatgccaccatgagctatgaatgccctatgctggatgaggggggtggaaccagatgacgtcgattgtt
ggtgcaacacgacgtcaacttgggttgtgtacggaacctgccatcacaaaaaaggtgaagcacggagatctagaagagctgtgacgctcccctcccattccactaggaagctgcaaacgcggtcgcaaacctggt
tggaatcaagagaatacacaaagcacttgattagagtcgaaaattggatattcaggaaccctggcttcgcgttagcagcagctgccatcgcttggcttttgggaagctcaacgagccaaaaagtcatatacttgg
tcatgatactgctgattgccccggcatacagcatcCGgtgcatCGgCGtaagcaacagagatttCGtCGaaggcatgtCGggCGgcaCGtgggttgaCGtCGtcttggaacaCGgaggttgtgtaaCGgtcatgg
cacaagacaaacccaCGgttgacatagaacttgtgaCGacaacCGtcagcaacatggcCGaagttCGatcttactgctatgaagcctccatttcCGacatggcatctgatagtCGatgtcCGaCGcaaggCGaag
catatcttgacaaacaatCGgacactcaataCGtatgcaaaCGaaCGttagtggatCGaggatggggaaaCGgatgCGgactattCGgaaaagggagtctCGtaaCGtgtgctaaattCGCGtgttCGaaaaaaa
tgaCGgggaagagcatacaaccagagaatctagaatacCGtatcatgctttCGgttcaCGgatctcaacacagCGgaatgatCGtgaatgacaCGggacatgaaaCGgaCGaaaatCGCGCGaaagttgagataa
ctcctaattcacctCGagCGgaagcaaCGctCGgtggtttCGgaagtcttggactagattgtgaacCGCGaacCGgtctCGatttttCGgatttgtattatttgaCGatgaacaacaagcattggttggttcaca
aagaatggtttcaCGacattccattgccttggcatgCGggCGCGgataCGggtaCGcCGcattggaacaacaaagaagcactagttgagttcaaagaCGcacatgCGaaaCGtcaaacagtCGtagtcctaggga E
gtcaagaaggCGCGgttcacacCGCGctCGcCGgCGCGctagaagctgagatggaCGgCGCGaagggaCGactgtCGtctggtcatttgaaatgtCGactgaaaatggacaaacttCGattaaaggtgtatcat
attCGttgtgcaCGgCGgctttcactttcacaaagatccCGgcagagacacttcaCGgcacCGtgacCGtCGaggtccaatatgCGggcacCGaCGgacCGtgtaaagttccCGCGcagatggcCGtggacatgc
aaaCGctgaCGccagtCGgtaggttgatcaCGgCGaatcCGgtgatcaCGgaaagcaCGgaaaactCGaagatgatgctagaacttgatcCGccattCGgagactcttatattgtaattggCGtaggCGagaaaa
aaataaCGcatcattggcatCGaagCGggagcaCGattggaaaagcatttgaagCGactgttCGaggCGcaaaaCGaatggcagttttgggCGacacCGCGtgggacttCGgatcagtCGgCGgagcactgaatt
CGttgggaaaaggaattcatcaaattttCGgCGcagCGttcaaatcattgtttggaggaatgtCGtggttttcacaaattctcataggaaCGttgctgatgtggttgggtctgaacacaaagaaCGgatcaattt
CGctcatgtgtttagCGttgggCGgtgtgttgatattttgtcaactgCGgtttctgctgatgtgggatgttCGgtagattttCGaaaaaagaaaCGCGatgCGgtacCGgagtgtttgtttacaaCGaCGtCG
aagCGtggCGCGatCGttacaaatatcatccCGattCGcCGCGtCGattggcCGcagcCGtgaagcaagcatgggaagaCGgcatctgCGggatttcatcCGtttCGCGaatggaaaacataatgtggCGatcCG
tCGaaggagaactaaatgCGatcctggaagaaaaCGgggttcaactgacCGtggtggtCGgatCGgtgaaaaatcCGatgtggCGCGgtcCGcagCGattgccCGttccCGtaaatgaacttcCGcatggatgga
aagcttggggaaatCGtatttCGtgCGCGcCGcaaagacaaacaacagtttCGttgtggaCGgagataCGctgaaagaatgccCGctgaaacatCGtgCGtggaatagttttctggtggaagatcaCGgtttCG NS1
gCGtatttcataCGagCGtgtggctgaaagttCGCGaagattattCGttggaatgtgatcCGgcagttattggaacCGCGgtgaaaggaaaagaagcCGttcacagtgatctaggatactggatCGaaagCGaaa
aaaaCGacaCGtggCGgctgaaaCGCGCGcatctgatCGagatgaaaaCGtgCGaatggcCGaaatCGcacaCGttgtggacCGaCGgaatCGaagagagCGatctgatcattcCGaaatcattagcCGgtcCGc
tcagtcatcataataCGCGCGaaggatatCGaactcaaatgaaaggacCGtggcacagCGaagaactagaaattCGtttCGaagaatgtccCGgaaccaaggtgcaCGtggaggaaacatgtggaacaagaggac
catctctgagatcaaccactgcaagCGgaagggtgatCGaggaatggtgctgcagggagtgcacaatgccccccactgtCGttcCGggctaaagatggctgttggtatggaatggagataaggcccaggaaagaac
cagaaagtaacttagtaaggtcaatggtgactgcaggatcaactgatcacatggatcacttctcccttggagtgcttgtgattctgctcatggtgcaggaagggctgaagaagagaatgaccacaaagatcatca
taagcacatcgatggcagtgctggtagctatgatcctgggaggattttcaatgagtgacctggctaagcttgcaattttgatgggtgccaccttcgcggaaatgaacactggaggagatgtagctcatctggcgc
tgatagcggcattcaaagtcagaccagcgttgctggtatctttcatcttcagagctaattggacaccccgtgaaagcatgctgctggccttggcctcgtgtcttttgcaaactgcgatctccgccttggaaggcg
acctgatggttctcatcaatggttttgctttggcctggttggcaatacgagcgatggttgttccacgcactgataacatcaccttggcaatcctggctgctctgacaccactggcccggggcacactgcttgtgg
cgtggagagcaggccttgctacttgcggggggtttatgctcctctctctgaagggaaaaggcagtgtgaagaagaacttaccatttgtcatggccctgggactaaccgctgtgaggctggtcgaccccatcaacg
tggtgggactgctgttgctcacaaggagtgggaagcggagctggcccccctagcgaagtactcacagctgttggcctgatatgcgcattggctggagggttcgccaaggcagatatagagatggctgggcccatgg
ccgcggtcggtctgctaattgtcagttacgtggtctcaggaaagagtgtggacatgtacattgaaagagcaggtgacatcacatgggaaaaagatgcggaagtcactggaaacagtccccggctcgatgtggcgc
tagatgagagtggtgatttctccctggtggaggatgacggtcccccatgagagagatcatactcaaggtggtcctgatgaccatctgtggcatgaacccaatagccataccctttgcagctggagcgtggtacg
tatacgtgaagactggaaaaaggagtggtgctctatgggatgtgcctgctcccaaggaagtaaaaaaggggagaccacagatggagtgtacagagtaatgactcgtagactgctaggttcaacacaagttggag
tgggagttatgcaagagggggtctttcacactatgtggcacgtcacaaaaggatccgcgctgagaagcggtgaagggagacttgatccatactggggagatgtcaagcaggatctggtgtcatactgtggtccat
ggaagctagatgccgcctgggacgggcacagcgaggtgcagctcttggccgtgccccccggagagagagcgaggaacatccagactctgcccggaatatttaagacaaaggatggggacattggagcggttgcgc
tggattacccagcaggaacttcaggatctccaatcctagacaagtgtgggagagtgataggactttatggcaatggggtcgtgatcaaaaatgggagttatgttagtgccatcacccaagggaggagggaggaag
agactcctgttgagtgcttcgagccttcgatgctgaagaagaagcagctaactgtcttagacttgcatcctggagctgggaaaaccaggagagttcttcctgaaatagtccgtgaagccataaaaacaagactcc
gtactgtgatcttagctccaaccagggttgtcgctgctgaaatggaggaagcccttagagggcttccagtgcgttatatgacaacagcagtcaatgtcacccactctggaacagaaatcgtcgacttaatgtgcc
atgccaccttcacttcacgtctactacagccaatcagagtccccaactataatctgtatattatggatgaggcccacttcacagatccctcaagtatagcagcaagaggatacatttcaacaagggttgagatgg
gcgaggcggctgccatcttcatgaccgccacgccaccaggaacccgtgacgcatttccggactccaactcaccaattatggacaccgaagtggaagtcccagagagagcctggagctcaggctttgattgggtga
cggatcattctggaaaaacagtttggtttgttccaagcgtgaggaacggcaatgagatcgcagcttgtctgacaaaggctggaaaacgggtcatacagctcagcagaaagacttttgagacagagttccagaaaa
caaaacatcaagagtgggactttgtcgtgacaactgacatttcagagatgggcgccaactttaaagctgaccgtgtcatagattccaggagatgcctaaagccggtcatacttgatggcgagagagtcattctgg
ctggacccatgcctgtcacacatgccagcgctgcccagaggagggggcgcataggcaggaatcccaacaaacctggagatgagtatctgtatggaggtgggtgcgcagagactgacgaagaccatgcacactggc
ttgaagcaagaatgctccttgacaatatttacctccaagatggcctcatagcctcgctctatcgacctgaggccgacaaagtagcagccattgagggagagttcaagcttaggacggagcaaaggaagacctttg
tggaactcatgaaaagaggagatcttcctgtttggctggcctatcaggttgcatctgccggaataacctacacagatagaagatggtgctttgatggcacgaccaacaacaccataatggaagacagtgtgccgg
cagaggtgtggaccagacacggagagaaaagagtgctcaaaccgaggtggatggacgccagagtttgttcagatcatgcggccctgaagtcattcaaggagtttgccgctgggaaaagaggagcggcttttggag
tgatggaagccctgggaacactgccaggacacatgacagagagattccaggaagccattgacaacctcgctgtgctcatgcgggcagagactggaagcaggccttacaaagccgcggcggcccaattgccggaga
ccctagagaccattatgcttttggggttgctgggaacagtctcgctgggaatctttttcgtcttgatgaggaacaagggcatagggaagatgggctttggaatggtgactcttggggccagcgcatggctcatgt
ggctctcggaaattgagccagccagaattgcatgtgtcctcattgttgtgttcctattgctggtggtgctcatacctgagccagaaaagcaaagatctccccaggacaaccaaatggcaatcatcatcatggtag
cagtaggtcttctgggcttgattaccgccaatgaactcggatggttggagagaacaaagagtgacctaagccatctaatgggaaggagagaggagggggcaaccataggattctcaatggacattgacctgcggc
cagcctcagcttgggccatctatgctgccttgacaactttcattaccccagccgtccaacatgcagtgaccacttcatacaacaactactccttaatggcgatggccacgcaagctggagtgttgtttggtatgg
gcaaagggatgccattctacgcatgggactttggagtcccgctgctaatgataggttgctactcacaattaacacccctgaccctaatagtggccatcattttgctcgtggcgcactacatgtacttgatcccag
ggctgcaggcagcagctgcgcgtgctgcccagaagagaacggcagctggcatcatgaagaaccctgttgtggatggaatagtggtgactgacattgacacaatgacaattgacccccaagtggagaaaaagatgg
gacaggtgctactcatagcagtagccgtctccagcgccatactgtcgcggaccgcctggggggtgggggaggctggggccctgatcacagcggcaacttccactttgtgggaaggctctccgaacaagtactgga
actcctctacagccacttcactgtgtaacattttagggggaagttacttggctggagcttctctaatctacacagtaacaagaaacgctggcttggtcaagagacgtgggggtggaacaggagagaccctgggag
agaaatggaaggcccgcttgaaccagatgtcggccctggagttctactcctacaaaaagtcaggcatcaccgaggtgtgcagagaagaggcccgccgcgccctcaaggacggtgtggcaacgggaggccatgctg
tgtcccgaggaagtgcaaagctgagatggttggtggagcggggatacctgcagccctatggaaaggtcattgatcttggatgtggcagaggggggctggagttactacgccgccaccatccgcaaagttcaagaag
tgaaaggatacacaaaaggaggccctggtcatgaagaacccatgttggtgcaaagctatgggtggaacatagtccgtcttaagagtggggtggacgtctttcatatggcggctgagccgtgtgacacgttgctgt
gtgacataggtgagtcatcatctagtcctgaagtggaagaagcacggacgctcagagtcctctccatggtggggggattggcttgaaaaaagaccaggagccttttgtataaaagtgttgtgcccatacaccagca
ctatgatggaaaccctggagcgactgcagcgtaggtatgggggaggactggtcagagtgccactctcccgcaactctacacatgagatgtactgggtctctggagcgaaaagcaacaccataaaaagtgtgtcca
ccacgagccagctcctcttggggcgcatggacggcccaggaggccagtgaaatatgaggaggatgtgaatctcggctctggcacgcgggctgtggtaagctgcgctgaagctcccaacatgaagatcattggta
accgcattgaaaggatccgcagtgagcacgcggaaacgtggttctttgacgagaacccacccatataggacatgggcttaccatggaagctatgaggcccccacacaaggtcagcgtcctctctaataaacgggg
ttgtcaggctcctgtcaaaaccctgggatgtggtgactggagtcacaggaatagccatgaccgacaccacaccgtatggtcagcaaagagttttcaaggaaaaagtggacactagggtgccagacccccaagaag
gcactcgtcaggttatgagcatggtctcttcctggttgtggaaagagctaggcaaacacaaacggccacgagtctgtaccaaagaagagttcatcaacaaggttcgtagcaatgcagcattaggggcaatatttg
aagaggaaaaagagtggaagactgcagtggaagctgtgaacgatccaaggttctgggctctagtggacaaggaaagagagcaccacctgagaggagagtgccagagttgtgtgtacaacatgatgggaaaaagag
aaaagaaacaagggaatttggaaaggccaagggcagccgcgccatctggtatatgtggctaggggctagatttctagagttcgaagcccttggattcttgaacgaggatcactggatggggagagagaactcag
gaggtggtgttgaagggctgggattacaaagactcggatatgtcctagaagagatgagtcgcataccaggaggaaggatgtatgcagatgacactgctggctgggacacccgcatcagcaggtttgatctggaga
atgaagctctaatcaccaaccaaatggagaaagggcacagggccttggcattggccataatcaagtacacataccaaaacaaagtggtaaaggtccttagaccagctgaaaaaggaagacagttatggacatta
tttcgagacaagaccaaaggggggagcggacaagttgtcacttacgctcttaacacatttaccaacctagtggtgcaactcattcggaatatggaggctgaggaagttctagagatgcaagacttgtggctgctgc
ggaggtcagagaaagtgaccaactggttgcagagcaacggatgggataggctcaaacgaatggcagtcagtggagatgattgcgttgtgaagccaattgatgataggtttgcacatgccctcaggttcttgaatg
atatgggaaaagttaggaaggacacacaagagtggaaaccctcaactggatgggacaactgggaagaagttccgttttgctcccaccacttcaacaagctccatctcaaggacgggaggtccattgtggttccct
gccgccaccaagatgaactgattggccgggcccgcgtctctccaggggcgggatggagcatccgggagactgcttgcctagcaaaatcatatgcgcaaatgtggcagctcctttatttccacagaagggacctcc
gactgatggccaatgccatttgttcatctgtgccagttgactgggttccaactgggagaactacctggtcaatccatggaaagggagaatggatgaccactgaagacatgcttgtggtgtggaacagagtgtgga
ttgaggagaacgaccacatggaagacaagaccccagttacgaaatggacagacattccctatttgggaaaaagggaagacttgtggtgtggatctctcatagggcacagaccgcgcaccacctggctgagaaca
ttaaaaacacagtcaacatggtgcgcaggatcataggtgatgaagaaaagtacatggactacctatccacccaagttcgctacttgggtgaagaagggtctacacctggagtgctgtaagcaccaatcttagtgt
tgtcaggcctgctagtcagccacagcttggggaaagctgtgcagcctgtgacccccccaggagaagctgggaaaccaagcctatagtcaggccgagaacgccatggcacggaagaagccatgctgcctgtgagcc
cctcagaggacactgagtcaaaaaaccccacgcgcttggaggcgcaggatggaaaagaaggtggcgaccttccccacccttcaatctggggcctgaactggagatcagctgtggatctccagaagagggactag
tggttagaggagacccccggaaaacgcaaaacagcatattgacgctgggaaagaccagagactccatgagtttccaccacgctggccgccaggcacagatcgccgaatagcggcggccggtgtggggaaatcca
tgggtct
```

Supplementary Figure 3 (continued).

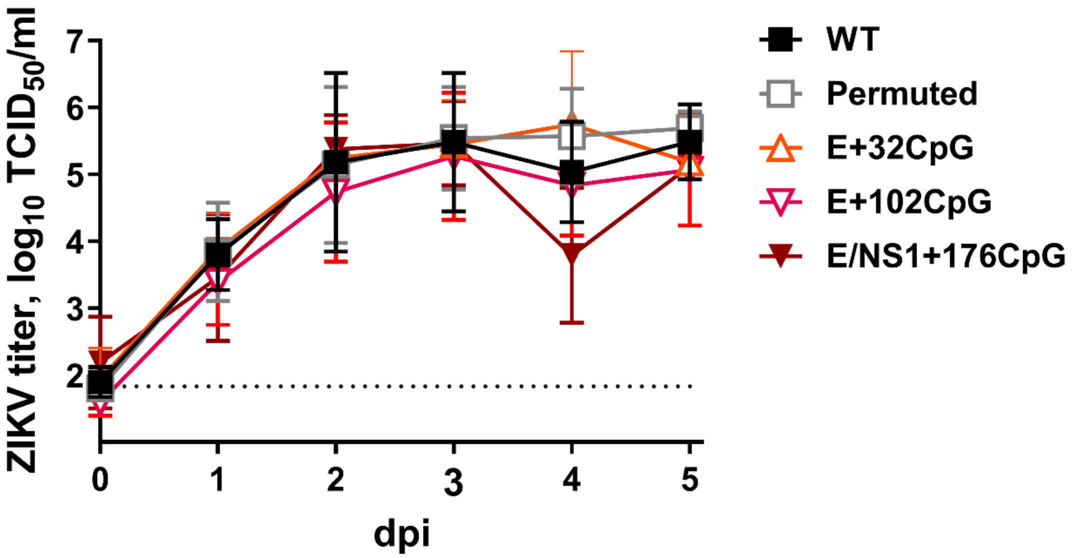

Supplementary Figure 4. Virus infection kinetics in C6/36 cells after inoculation at a multiplicity of infection (MOI) of 0.01. Cell culture supernatants were collected and viral titers were measured using the endpoint dilution assay. The dotted horizontal line represents the limit of detection. Whiskers represent standard error of the mean (SE) from three biologically independent replicates. “dpi:” days post inoculation. Dunnett’s test showed no multiplicity-adjusted statistically significant p values for all variants versus WT.
