# Supplementary material for: CpG-Recoding in Zika Virus Genome Causes Host-Age-Dependent Attenuation of Infection With Protection Against Lethal Heterologous Challenge in Mice
Source: Front Immunol. 2020 Jan 24;10:3077. doi: 10.3389/fimmu.2019.03077 (PMC6993062; doi:10.3389/fimmu.2019.03077)
Supplement: Supplementary file 2 [file Data_Sheet_2.PDF]

Supplementary Material

Supplementary Table 1. Primers used for amplification and sequencing of recoded ZIKV fragments for *in vitro* stability assay.

Primers	Genomic region	Genomic nucleotide #	Primer sequence 5'-3'	ZIKV variants
ZIKV-F919[a]	M	919-945	CAACGAGCCAAAAAGTCATATACTTGG	all
ZIKV-F1416	E	1416-1435	CAGCACAGTGGGATGATCGT	WT<br>Permuted<br>E+32CpG
ZIKV-F1416-bis	E	1416-1435	CAACACAGCGGAATGATCGT	E+102CpG<br>E/NS1+176CpG
ZIKV-F2007[b]	E	2007-2025	CAGATGGCGGTGGACATGC	all
ZIKV-R2026	E	2010-2026	TGCATGTCCACCGCCAT	all
ZIKV-R2370[b]	E	2349-2370	GTGAGAACCAGGACATTCCTCC	all
ZIKV-R3498[b]	NS1	3477-3498	GCCTTATCTCCATTCCATACCA	all
ZIKV-R3960[a,b]	NS2A	3943-3960	TTGCCAACCAGGCCAAAG	all

[a] ZIKV-F919 and ZIKV-R3960 primers were used to amplify the 3-kb DNA fragment containing sequences encoding the E (978 … 2489 = 1512 nt) and NS1 (2490 … 3545 = 1056 nt) proteins.
[b] [Weger-Lucarelli J, *et al*. Rescue and characterization of recombinant virus from a New World Zika virus infectious clone. *J Vis Exp* (2017) **124**:e55857. doi:10.3791/55857].

Supplementary Table 2. Primers used for amplification and sequencing of recoded ZIKV fragments for *in vivo* stability assay.

Primers	Genomic region	Genomic nucleotide #	Primer sequence 5'-3'	ZIKV variants
ZIKV-F658[a]	pr	658-680	CAGATGACGTCGATTGTTGGTGC	E/NS1+176CpG
ZIKV-F919[b]	M	919-945	CAACGAGCCAAAAAGTCATATACTTGG	E+32CpG<br>E+102CpG
ZIKV-F1321	E	1321-1343	CGTGTGCTAAATTCGCGTGTTCG	E/NS1+176CpG
ZIKV-F2042[a]	E	2042-2060	CGGTAGGTTGATCACGGCG	E+32CpG<br>E+102CpG<br>E/NS1+176CpG
ZIKV-R2296[a]	E	2277-2296	GAATTCAGTGCTCCGCCGAC	E+32CpG<br>E+102CpG<br>E/NS1+176CpG
ZIKV-R2539[b]	NS1	2519-2539	GTACCGCATCTCGTCTCCTTC	E+32CpG<br>E+102CpG
ZIKV-R3762[a]	NS1	3741-3762	CTCCTCCAGTGTTCATTTCCGC	E/NS1+176CpG

[a] For sequencing of the E/NS1+176CpG ZIKV variant two sets of primers (ZIKV-F658 + ZIKV-R2296 and ZIKV-F2042 + ZIKV-R3762) were used to amplify the two DNA fragments containing sequences encoding the E (978 … 2489 = 1512 nt) and NS1 (2490 … 3545 = 1056 nt) proteins.
[b] For sequencing of the E+32CpG and E+102CpG ZIKV variants ZIKV-F919 and ZIKV-R2539 primers were used to amplify the 1.6-kb DNA fragment containing the sequence encoding the E protein (978 … 2489 = 1512 nt).
